# Gene set enrichment analysis for non-monotone association and multiple experimental categories

**DOI:** 10.1186/1471-2105-9-481

**Published:** 2008-11-14

**Authors:** Rongheng Lin, Shuangshuang Dai, Richard D Irwin, Alexandra N Heinloth, Gary A Boorman, Leping Li

**Affiliations:** 1 Biostatistics Branch, National Institute of Environmental Health Science, Research Triangle Park, NC 27713, USA; 2 Alpha-Gamma Technologies, Inc., Raleigh NC 27609, USA; 3 Environmental Toxicology Program, National Institute of Environmental Health Science, Research Triangle Park, NC 27713, USA; 4 Laboratory of Molecular Toxicology, National Institute of Environmental Health Science, Research Triangle Park, NC 27713, USA; 5 Covance Inc., Vienna, VA 22066, USA

## Abstract

**Background:**

Recently, microarray data analyses using functional pathway information, e.g., gene set enrichment analysis (GSEA) and significance analysis of function and expression (SAFE), have gained recognition as a way to identify biological pathways/processes associated with a phenotypic endpoint. In these analyses, a local statistic is used to assess the association between the expression level of a gene and the value of a phenotypic endpoint. Then these gene-specific local statistics are combined to evaluate association for pre-selected sets of genes. Commonly used local statistics include t-statistics for binary phenotypes and correlation coefficients that assume a linear or monotone relationship between a continuous phenotype and gene expression level. Methods applicable to continuous non-monotone relationships are needed. Furthermore, for multiple experimental categories, methods that combine multiple GSEA/SAFE analyses are needed.

**Results:**

For continuous or ordinal phenotypic outcome, we propose to use as the local statistic the coefficient of multiple determination (i.e., the square of multiple correlation coefficient) *R*^2 ^from fitting natural cubic spline models to the phenotype-expression relationship. Next, we incorporate this association measure into the GSEA/SAFE framework to identify significant gene sets. Unsigned local statistics, signed global statistics and one-sided p-values are used to reflect our inferential interest. Furthermore, we describe a procedure for inference across multiple GSEA/SAFE analyses. We illustrate our approach using gene expression and liver injury data from liver and blood samples from rats treated with eight hepatotoxicants under multiple time and dose combinations. We set out to identify biological pathways/processes associated with liver injury as manifested by increased blood levels of alanine transaminase in common for most of the eight compounds. Potential statistical dependency resulting from the experimental design is addressed in permutation based hypothesis testing.

**Conclusion:**

The proposed framework captures both linear and non-linear association between gene expression level and a phenotypic endpoint and thus can be viewed as extending the current GSEA/SAFE methodology. The framework for combining results from multiple GSEA/SAFE analyses is flexible to address practical inference interests. Our methods can be applied to microarray data with continuous phenotypes with multi-level design or the meta-analysis of multiple microarray data sets.

## Background

Microarray technology profiles the expression levels of thousands of genes simultaneously, providing a snapshot of transcript levels in the cells/tissues being studied. Analysis of microarray data typically involves examining thousands of genes with relatively small sample sizes, and thus is challenging for statisticians [1,2]. Moreover, deriving useful biological knowledge from these gene sets is often a long and arduous task. Recently, however, methods have been developed that incorporate existing knowledge of biological pathways/processes into the analysis. For example, gene set enrichment analysis (GSEA) tests whether a known set of genes is associated with a phenotypic difference [3]. In GSEA, an enrichment score (ES) for a predefined gene set, usually a known biological pathway/process, is calculated and statistical significance is evaluated. GSEA was refined by [4] and a more general framework, significance analysis of function and expression (SAFE) was proposed by [5]. Furthermore, ES vectors for gene sets can be defined for individual samples and used for classification [6]. In these procedures, an association measure between a phenotypic endpoint and expression levels is calculated and subsequently used as the basis for further evaluation of the association between gene sets and the same phenotypic endpoint. Additional work on GSEA type methods can be found in [7-11].

Numerous statistics have been proposed to identify differentially expressed genes when the phenotype is binary [12-17]. All these statistics can be used to measure the association between gene expression levels and a binary phenotype. When a phenotypic endpoint is continuous, correlation analysis between gene expression and the phenotypic variable emerges naturally. A correlation measurement needs to be selected based on the goal of the investigation, biological interpretation, statistical properties and computational feasibility. The commonly used Pearson product-moment correlation coefficient measures linear association, whereas the non-parametric Spearman correlation measures monotone trend. A brief summary of several commonly used correlation measurements and their limitations can be found in [18]. Alternatively, the standardized Z-type statistics resulting from univariate Cox proportional hazard model has been proposed as the local statistics for the situation where continuous survival time with censoring is the phenotypic endpoint [5].

In biological research using microarray technology, gene expression data are often collected under multiple experimental conditions, along with traditional pathological endpoints [19]. One practical inferential goal is to identify biological pathways/processes that are associated with the endpoint and thus gain insight into biological mechanisms of tissue response to the experimental conditions. Under the framework of GSEA/SAFE, inference is based on assessing the association between expression levels of predefined sets of genes and the phenotypic endpoint. When associations between the phenotypic endpoint and the gene expressions are likely to be non-linear or non-monotone, the local statistics, which is the measurement of gene specific association to the endpoint, should be able to capture these, while a linear or monotone situation should still be accommodated. We propose to use the coefficient of multiple determination *R*^2 ^from a natural cubic spline model [20]. The spline model allows us to capture potential non-linear and non-monotone trends. The quantity *R*^2 ^is then used as the correlation in GSEA/SAFE to identify gene sets that are associated with the phenotypic endpoints.

When experimental conditions include multiple categories, different biological mechanisms might be expected and separate association analyses for each category are desired before aggregating to arrive at the final conclusion. We carry out separate enrichment analysis in each category and propose an *ad hoc *procedure to combine the results.

In this article, the words "association" and "correlation" are used interchangeably in most situations.

## Results and discussion

We describe the methods for separate analysis in each category in Section 2.1 and 2.2, then inference across categories in Section 2.3. The methods are then illustrated using National Center of Toxicogenomics (NCT) compendium data in Section 2.4.

The relevant R code is available at .

### 2.1 Non-linear association measurement

In all methods for the evaluation of gene sets discussed in the introduction, the association measure between a gene set and an endpoint is built from the association measures between individual genes and the endpoint. We would like a gene specific association measure to have several properties. First, it should be scale free, i.e., it would not change when data are linearly transformed. This property guarantees that in data preprocessing, different bases in log transformation (e.g., log_2 _or log_10_) give the same inferential conclusion. Second, it should accommodate non-linear or non-monotone associations between variables. Third, it should allow limited data points. Fourth, it should be easy to compute and interpret. These considerations exclude the usage of many non-linear correlation functions and measures, e.g., [21]. We propose to use the coefficient of multiple determination *R*^2 ^in natural cubic spline regression models to measure the gene specific association. This quantity can be interpreted as the fraction of the variation in endpoints that can be explained by the gene expression data. When the association is linear, this quantity is approximately equal to the square of Pearson correlation.

For experimental category *c*, *c *= 1, 2, ⋯, *C*, we denote $Y_{i}^{c}$ as the continuous or ordinal endpoint measurement of sample *i*, and $X_{ij}^{c}$ as the expression level of gene *g*_*j *_in sample *i*. The respective experimental condition of sample *i *is denoted as $e_{i}^{c}$. The category can be defined by tumor types, compounds, etc., and $e_{i}^{c}$ can contain other experimental factors, e.g., time and dose. Let $\epsilon_{i}^{c}$ be the random noise satisfying E[$\epsilon_{i}^{c}$] = 0. For convenience, the superscript *c *is suppressed in *X*, *Y*, *e *and *ε *without causing confusion in the sequel. We assume equation (1), i.e., the endpoint depends on experiment conditions in a way that is functionally related to gene expression levels:

(1) $$Y_{i}|X_{ij},g_{j},e_{j},\epsilon_{ij}=Y_{i}|X_{ij},g_{j},\epsilon_{ij}=f_{c,g_{j}}(X_{ij})+\epsilon_{ij}$$

We define

(2) $$R_{c,j}^{2}=R^{2}(Y,X_{j})=1-\frac{\sum_{i=1}^{n}{\left(y_{i}-{\hat{y}}_{i}(x_{ij})\right)}^{2}}{\sum_{i=1}^{n}{\left(y_{i}-\hat{Y}\right)}^{2}}$$

to assess the association between **X**_*j *_and **Y**, where $\hat{y}(x_{ij})={\hat{f}}_{c,g_{j}}(x_{ij})$ can be estimated by regression method accommodating possible non-linear trends. Here we use natural cubic splines, which are cubic splines with linear extension out of the boundary knots [20,22]. This flexible approach guarantees that both non-linear and linear trends are well captured. When a linear trend is the only desired type of association, we can simply change the splines into linear terms and the proposed methods can still be used.

The calculation of the quantity $R_{c,j}^{2}$ has two main steps:

• Specify inside knots at *b *evenly spread quantiles of **X**_*j*_. Generate B-spline basis matrix for natural cubic spline *B*(*X*_*j*_)_*n *× (*b*+2) _from **X**_*j *_(*b *+ 1 spline terms and one intercept term). We used *ns() *function in the *splines *package of *R *[23].

• Regress **Y **on *B*(**X**_*j*_) and obtain the $R_{c,j}^{2}$ value. We use *QR *decomposition routine *qr() *in *R *directly to avoid extraneous computation for other statistics such as coefficient estimates, standard errors, etc., that would occur if using the linear model regression routine *lm()*.

The piecewise cubic splines approach, unlike the regular *b *+ 1 degree polynomials, automatically provides the roughness penalty [24], which control the degree of ripples in fitted curve. Gene specific quantiles are easy to compute and guarantee enough sample points between knots. Some alternative ways, e.g., using global quantiles of all **X**_*j*_, do not have this property and would make poor fitting for some genes. For the number of knots to be used in splines model, "a minimum of 5 points should be between knot locations" has been suggested [25]. The selection of *b *depends on the available sample size and desired flexibility in capturing non-linear trend.

### 2.2 Adapted GSEA procedure

Given a gene set in an array, there have been two types of set specific global statistics [8]: "self-contained" one using only genes in the set and "competitive" one using all genes in the array. Approaches proposed in [26,27] belong to the first type and GSEA/SAFE approach belongs to the second type. Computationally, when there are only a small number of gene sets to test, "self-contained" approaches are much faster by avoiding the genes out of the sets.

GSEA method is designed to test whether a *pre-built *gene set is associated with the phenotype. Its kernel Kolmogorov-Smirnov statistic detects the difference of gene positions in the ranked gene list between one gene set and its complementary set. The method can thus highlight a gene set that has similar associations (and ranks) for each gene in the set. When the identified set of genes cluster close to the bottom of the ranked list ordered by association strength, the set is likely to be of limited interest to biologists [27-29]. Also, the method is biased toward those well-studied gene sets [8].

Belonging to the "self-contained" type, Goeman et al. (2004)'s approach [26] proposed to fit a random effect model for each gene set and has the potential to accommodate non-linear terms conveniently. It assumed the coefficients of genes are independent and otherwise a proper covariance matrix needs to be set up. When used with non-linear terms, the independence assumption thus need to be justified or more exploration on the proper covariance matrix specification will be expected. Once independence assumption holds or proper covariance matrix can be specified, the approach will be very attractive in computation.

GSEA/SAFE (and SAM-GS [27]) calculate one measurement for each gene and then for each gene set, accumulate the genes' signals into one for the whole gene set. For each gene, there is only one association measurement. Goeman et al. (2004) fits models directly for each gene set. The coefficients of the same gene in different gene sets could then be different and do not have direct interpretation of association strength, while there exist many other advantages for "self contained" approaches [8].

We construct the global statistics measuring the association between the phenotype and the pre-built sets of genes based on $R_{c,j}^{2}$ as local statistics. We adapt the modified version of GSEA [4] where genes with stronger association are assigned higher weight. The method does not require the independence assumption between genes. In each category *c *we define enrichment score (ES) for gene set *s*:

• Rank all *J *genes to form list *L *= {*g*_1_, ..., *g*_*J*_} decreasing in $R_{c,j}^{2}$.

• Evaluate the fraction of genes in *s *("hits") weighted by $R_{c,j}^{2}$ and the fraction of genes not in *s *("misses") present up to a given position *k *in *L*.

$$\begin{array}{ccc} P_{hit}(s,c,k) & = & \sum_{g_{j}\in s,j\leq k}\frac{R_{c,j}^{2}}{N_{R}},\text{where }N_{R}=\sum_{g_{j}\in s}R_{c,j}^{2},\\ P_{miss}(s,c,k) & = & \sum_{g_{k}\notin s,j\leq k}\frac{1}{J-N_{s}},\text{where }N_{s}\text{ is the number of genes in }s. \end{array}$$

• ES(s, c) is the *signed *maximum deviation from zero of *P*_*hit*_(*s*, *c*, *k*) - *P*_*miss*_(*s*, *c*, *k*) over *k*.

For a ranked gene list based on $R_{c,j}^{2}$, our inferential interest is on the top of the list, rather than both extremes as when ranking is based on t-statistics or Pearson correlations. This feature makes those sets with both positively and negatively correlated genes easier to identify than if using signed correlation measurements. Gene sets clustering at the top of the list will have positive maximum deviation and those clustering at the bottom of the list will have negative maximum deviation. Consequently, we use one sided p-values rather than two-sided p-values to indicate the significance of gene sets. A gene set cluster at the bottom of the list, which is off our interest, will have a p-value close to 1 and will not be highlighted. A signed local statistics, unsigned ES and two-side p-values were used in [4].

We calculate these p-values with permutations. We permute the phenotypic endpoint variable **Y **1000 times to generate 1000 permuted data sets and calculate the ES values for each permutation. These ES values form the null distribution of ES to estimate the p-value of the observed ES value. This permutation method retains the correlation structure among the genes. More discussion on the permutation details is deferred to Section 2.4.2.

### 2.3 Inference across experimental categories

In the standard framework of GSEA/SAFE, analysis is carried out only for one category. However, with multiple categories of experimental settings, "identifying biological pathways" that are associated with phenotypic changes in most categories" is a practically important inferential goal. When association patterns differ under different categories, pooling data is not a good option for either biological interpretation or statistical inference.

For a gene set *s*, separate analyses in *C *categories generate a p-value vector of length *C*. At first glance, Fisher's method of combining p-values [30] could be used along with false discovery rate (FDR) to select a threshold [31,32]. However, the goal of our method is to identify gene sets that are related to phenotypic changes for most of the categories, while Fisher's method could lead to very high significance even if strong association is observed in only one category. Therefore, we propose a subjective but stringent criterion to evaluate gene sets across categories.

Let *P*_*s*, *c *_be the p-value of set *s *for category *c*. To call one set significant across the *C *categories, we require at least *M *of *C *p-values *P*_*s*,1_, *P*_*s*,2_, ⋯, *P*_*s*, *C *_be less than *p*_0_, 1 ≤ *M *≤ *C*. In this study, we assume that the values of *M *and *C *are known fixed integers and will drop them from notations for convenience. Formally, the sets identified are defined as:

$$A(p_{0})=\{s:\sum_{c=1}^{C}I(P_{s,c}<p_{0})\geq M\}=\{s:P_{s,(M)}<p_{0}\}$$

where *P*_*s*,(*M*) _is the *M*^*th *^smallest of the *C *p-values *P*_*s*,1_, *P*_*s*,2_, ⋯, *P*_*s*, *C*_. By adjusting the value of *p*_0_, we can control the number of sets that meet the criteria.

Under a *global null hypothesis*, the gene set is not associated with phenotypic changes in any category and $f_{c,g_{j}}(X_{ij})$ in equation (1) equal to zero for each *c*; all *C *p-values independently have a Uniform(0,1) distribution. A direct calculation of the type I error rate *α *based on the proposed rule, using *p*_0 _is

(3) $$\alpha (p_{0})\leq \sum_{m=M}^{C}\left(\begin{array}{c} C\\ m \end{array}\right){\left(p_{0}\right)}^{m}{\left(1-p_{0}\right)}^{C-m}$$

An example given in Section 2.4 calculates *α*(*p*_0_) in similar way for more complicated practical inferential goal. One benefit of this approach is that it does not require p-values in very high precision, avoiding computational burden in permutation based hypothesis testing. See Section 2.4.2 for further discussion.

For each *p*_0_, a conservative estimation of the FDR bound can be given by the definition [31]:

(4) $$FDR(p_{0})\leq \frac{S\times \alpha (p_{0})}{\left|A\right|},$$

where *S *is the number of gene sets and |*A*| is the cardinality of *A*. The bound is inflated because it assumes that none of *S *gene sets are associated with the phenotypic changes. When |*A| *= 0, i.e., none of the gene sets is identified, FDR is 0 by definition. Since in most of cases, *α*(*p*_0_) is so small that this bound is small enough for practical usage. When more advanced FDR control methods are desired, extra assumptions, e.g., the independence between gene sets might be needed.

We note that under the global null hypothesis, it is straightforward to show that random variable *P*_*s*,(*M*) _follows the *Beta*(*M *- 1, *C *- *M*) distribution. We can use p-values of observed *P*_*s*,(*M*) _to select gene sets and evaluate the type I error rate, which provides an equivalent alternative to equation (3). By this approach, the set-specific FDR can be calculated based on the empirical distribution of set-specific p-values [31,32].

One alternative approach for inference across categories is to control the category specific FDR and then combine the results. Depending on the goal, the unions or intersections of *C *lists of gene sets identified by proper FDR thresholds can be used. However, approaches to estimate the FDR usually require very precise estimation of p-values [33] and 1000 permutations can produce p-values with the granularity up to 0.001. Several methods have been proposed on this issue: assuming the normalized null of ES for different sets are the same and pooling them to obtain a global null distribution with much finer granularity [3,4]; using a linear combination of t-statistics as the global statistic, which asymptotically has normal distribution [7]; using more permutations (eg., 10,000 times) to generate a set-specific null distribution [5]. However, asymptotic results for *R*^2 ^(and thus ES) may not be available as discussed further in additional file 1, section 1. For a large data set with many categories, 10,000 permutations would constitute a substantial computational burden. More importantly, it is unclear how the category-wise FDRs should be combined into one interpretable summary statistic. To the best of our knowledge, no literature is available on this issue.

### 2.4 Analysis of NCT compendium data

The National Center for Toxicogenomics (NCT) compendium data were recently developed at National Institute of Environmental Health Science (NIEHS), NIH. Agilent cDNA oligonucleotide microarrays were used to profile the expression level of 20500 genes from both the liver and blood of male rats (Rattus norvegicus, F344/N strain) treated with 8 hepatotoxic compounds: bromobenzene, 1; 2-dichlorobenzene, 1; 4-dichlorobenzene, diquat dibromide (diquat), galactosamine, monocrotaline, n-nitrosomorpholine and thioacetamide, each at multiple time-dose combinations. These 8 compounds target different cell types and regions in the liver, and thus each compound in each tissue is considered a category. There were 4–6 replicates per condition and a total of 318 treated animals. Table 1 lists the data structure. Many pathological phenotypic endpoints were collected for the samples and we use a liver injury indicator, alanine transaminase (ALT) level as the outcome variable **Y **in the following analysis. More details on experimental design, histopathology, and clinical chemistry can be found in [34].

**Table 1:** NCT compendium microarray data. In time rows, the numbers from 0 to 3 indicate vehicle, low, medium and high doses.

Compound	1.2.Di	1.4.Di	Brom	Diqu	Gala	Mono	N-Nitr	Thio
6 Hrs	0–3	0–3	0–3	0–4	0–3	0–3	0–3	0–3
24 Hrs	0–3	0–3	0–3	0–4	0–3	0–3	0–3	0–3
48 Hrs	0–3*	0–3	0–3	0–4	0–3	0–2^†^	0–3	0–3
Replicates	4	4	4	6	4	4	4	4
Array totals	34	36	36	72	36	32	36	36

Diquat dibromide has 4 levels. Array numbers do not include vehicle treated animals, which were used as baseline in cDNA microarray. *: Two animals in high dose group died before 48 hours. †: All four animals in high dose group died before 48 hours.

**Figure 1:**
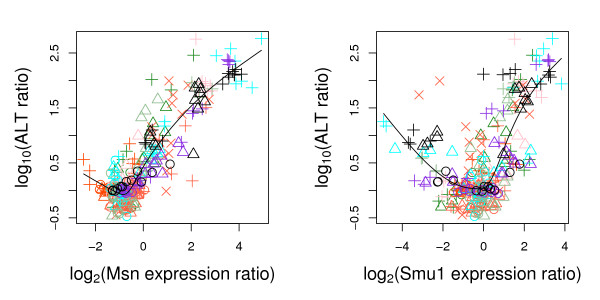
**log_10_(ALT ratio) vs log_2_(gene expression ratio).** Examples of non-monotone relationship in liver between expression levels of two genes and ALT. Each color represents one compound, and symbols (○, Δ, +) indicate the low, medium and high doses respectively. For the compound (diquat) with four dose levels, symbol "×" indicates the highest dose. Time information is not indicated. The smoothing line is fitted using all data, with natural cubic splines.

In microarray data analysis, expression ratios are often log transformed for analysis convenience. Since ALT levels range from generally less than 100 units (I.U/ml) in vehicle treated animals to several thousand units after exposure to a hepatotoxicant, log transformation of ALT levels is also necessary. In the following, we use gene expression level to refer to the log_2 _ratio of gene expression levels of treated rats to the control rats. Similarly, we use ALT level to refer to the log_10 _ratio of ALT levels of treated rats to the mean of those in respective group of vehicle treated rats (see Table 1). The different log transformation bases are for reading and presenting convenience only and do not change our inferential conclusion as noted in Section 2.1. Figure 1 show some examples of observed non-linear and non-monotone relationships between ALT level and gene expression levels. A smoothing line using all data is provided as baseline in the figure; the different compounds depart from this smooth line in different patterns, indicating compound specific spline fittings should be used.

We assume that the ALT depends on time and dose in a way that is functionally related to gene expression levels, i.e., *e*_*i *_in equation 1 contains time and dose information. We let *b*, the number of inside knots as described in Section 2.1, equal 4. It is close to the maximum number we can afford with 32–72 samples per compound, following the suggestion "a minimum of 5 points should be between knot locations" [25]. With the degrees of freedom equal 5 (4 inside knots), the model is flexible enough to capture major biologically plausible departures from linear or monotone trend. The same spline space has been used in [13] to calculate a gene's posterior "probability" of differential expression through logistic regression.

**Figure 2:**
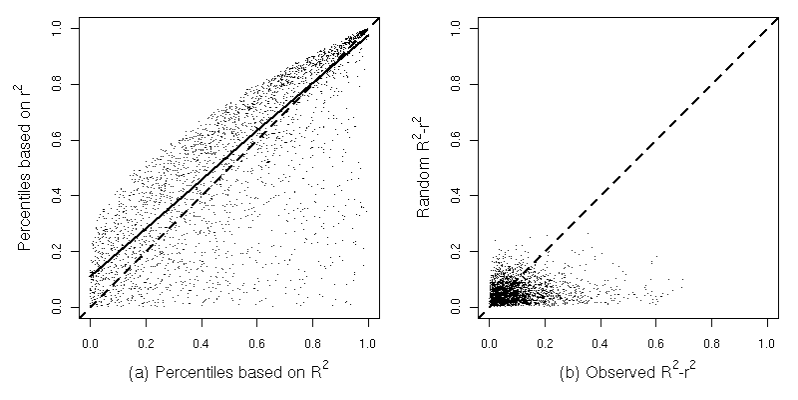
**(a) Genes' percentiles based on *R*^2 ^and the square of Pearson correlation (*r*^2^).** Gray dots show (*R*^2^, *r*^2^) and the solid line is a smoothing (lowess) line. The dashed line is *y *= *x*. (b) Observed *R*^2 ^- *r*^2 ^vs randomly generated *R*^2 ^- *r*^2^. The dashed line is *y *= *x*.

Without gold criteria for the performance improvement using the proposed splines model and *R*^2 ^in GSEA, we use Figure 2 as a qualitative support. we calculate the square of the Pearson correlation (*r*^2^) between ALT and each gene, which is equal to the *R*^2 ^from a simple linear model. We plot the 20500 genes' percentiles based on *r*^2 ^and on our proposed *R*^2 ^for one compound, diquat in liver (Figure 2, panel 1). The percentiles/ranks of genes are very important for identifying either differentially expressed genes or gene sets associated with phenotypic changes. The departure of the dots from the diagonal in the figure indicates a large difference in the percentiles/ranks based on the two quantities and the necessity of accommodating non-linear trends. To eliminate the possibility that the departure is simply due to the extra degrees of freedom in non-linear model, we randomly permuted the ALTs and calculated *R*^2 ^- *r*^2 ^for all genes (Figure 2, panel 2). The range of observed *R*^2 ^- *r*^2 ^is much wider than that of randomly permuted data set. For genes that have a large observed *R*^2 ^- *r*^2 ^values, their non-linear association is better captured by using *R*^2 ^and ranks would change greatly by using non-linear model. Similar patterns were observed for the other compounds in both tissues.

To build enrichment scores (ES) indicating the associations between ALT and gene sets from *R*^2^, pre-built gene sets are needed. A total of 466 pre-built gene sets are prepared with methods detailed in additional file 1, section 2. In our implementation, each compound is analyzed separately in each tissue. As described in Section 2.2, we obtain 466 p-values (one per gene set) for each of 8 compounds per tissue under the null hypothesis that the gene expression levels in the tissue are not associated with ALT for this compound.

Let $P_{s,c}^{A}$ be the p-value of set *s *for compound *c *in liver and $P_{s,c}^{B}$ in blood. To call one set significant, we require at least 6 of 8 (a subjective definition of "most") p-values $P_{s,1}^{L},P_{s,2}^{L},\cdots ,P_{s,8}^{L}$ be less than *p*_0_, i.e., *M *= 6, *C *= 8. In this article, we use *p*_0 _= 0.05 and 0:1 such that the number of identified gene sets are manageable.

Using formula (3),

*α*_*L*_(0.05) = *α*_*B*_(0.05) = 4.0 × 10^-7^, *α*_*L*_(0.1) = *α*_*B*_(0.1) = 2.3 × 10^-5^

To identify gene sets that are associated with ALT changes in both liver and blood, we require that at least 6 of 8 p-value pairs ($P_{s,c}^{A}$, $P_{s,c}^{B}$) satisfy that both values are less than *p*_0_.

$$A_{LB}(p_{0})=\{s:\sum_{c=1}^{8}I(P_{s,c}^{L}<p_{0}\text{ and }P_{s,c}^{B}<p_{0})\geq 6\}$$

By definition, we have *A*_*LB*_(*p*_0_) ⊆ *A*_*L*_(*p*_0_), *A*_*LB*_(*p*_0_) ⊆ *A*_*B*_(*p*_0_) and if *p*_0 _≤ ${p^{\prime }}_{0}$, *A*_*l*_(*p*_0_) ⊆ *A*_*l*_(${p^{\prime }}_{0}$), *l *= *L*, *B*, *LB*. Generally, we have

*α*_*LB*_(0.05) ≤ *α*_*L*_(0.05) = 4.0 × 10^-7^, *α*_*LB*_(0.1) ≤ *α*_*L*_(0.1) = 2.3 × 10^-5^

In NCT compendium data, no direct associations were observed between gene expressions in liver and in blood. This can due to different response mechanisms to stressors or time lag between liver and blood, which can not be verified with sparse time points. With this observation, we can further assume the independence of gene expressions between tissues. Under the null hypothesis that the gene set is not associated with liver injury for any compound and tissue, we have independence between the tissues and the compounds. Similar to formula (3), type I error rate is

$$\begin{array}{c} \alpha_{LB}(p_{0})\leq {\left(p_{0}^{2}\right)}^{8}+\left(\begin{array}{c} 8\\ 7 \end{array}\right)\times {\left(p_{0}^{2}\right)}^{7}\times (1-p_{0}^{2})+\left(\begin{array}{c} 8\\ 6 \end{array}\right)\times {\left(p_{0}^{2}\right)}^{6}\times {\left(1-p_{0}^{2}\right)}^{2}\\ \alpha_{LB}(0.05)=7\times 10^{15},\alpha_{LB}(0.1)=2.8\times 10^{-11} \end{array}$$

#### 2.4.1 Analysis results

In general, sets are more significant in liver than in blood. Using *p*_0 _= 0.05, we identified 38 gene sets (*A*_*L*_(0.05)) in liver and 2 gene sets (*A*_*B*_(0.05)) in blood. Five sets are identified for *A*_*LB*_(0.1) while none of them attain the criteria of *A*_*LB*_(0.05). The FDR bound (Equation 4) for all reported *A*_*l*_(*p*_0_), *l *= *L*, *B*, *LB*, *p*_0 _= 0.05, 0.1 are smaller than 4 × 10^-4^. Biological interpretation of these sets are provided in additional file 1, section 3.

We note that by setting *p*_0 _= 0.05 and 0:1, we have used a relatively conservative approach to select a small number of the most significant sets that represent good candidates for further study. For completeness, we provide all intermediate results from step 2 in the additional file 2 (and available upon request).

We also carried out a two-way (gene expression and ALT) clustering analysis using software *Cluster *[35] for each of the 5 sets from *A*_*LB*_(0.1). Heat maps are provided in the additional file 3. One can see that liver and blood have different patterns of gene expression, supporting our procedure of analyzing liver and blood separately. In addition, this difference suggests that it would be difficult to use blood gene expression to predict liver gene expression or vice versa in this data set. Some identified sets contain both genes positively or negatively correlated to ALT, indicating that in calculating ES values for general GSEA/SAFE procedure, genes should be ranked according the absolute value of their associations to the phenotypic endpoint.

**Figure 3:**
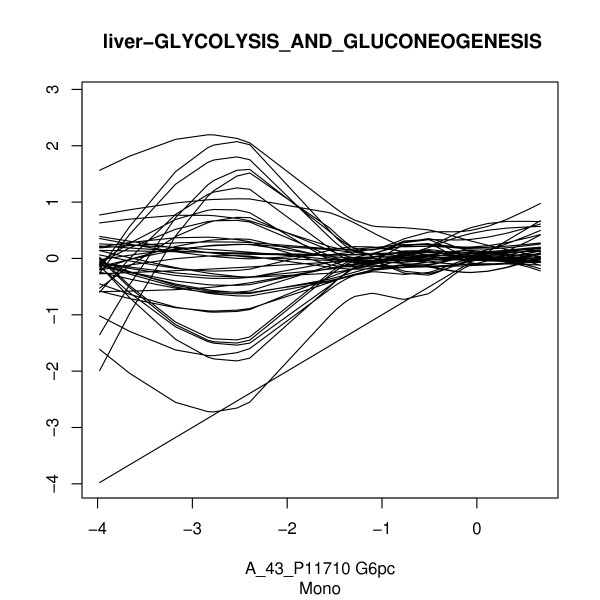
**Rich association types between genes in gene set *glycolysis and gluconeogenesis*.** X-axis is the expression level of the gene G6pc which has the largest standard deviation in the gene set. Y-axis is the expression level for all genes in the set. Natural cubic splines with 4 inner knots at quartiles are fitted using G6pc as the predictor variable. Data for monocrotaline in liver are used.

When using Pearson correlation to account for linear association, an identified set can have many genes linearly associated to the phenotype and so these associated genes are likely to associate each other linearly. We present in Figure 3 rich association relations between genes with one of identified sets, *glycolysis and gluconeogenesis *with liver gene expression data in monocrotaline. The figures for other compounds are provided in the additional file 4. The figures show that for the genes in the same biological pathway, linear functions are far from enough to describe the association patterns between genes. Both positive, negative and non-monotone association between genes are observed in the presented pathway. This observation indicates that we need to be careful in using signed association measurements to build global statistics. Rather than cumulating the association strength of genes in the set, it is possible that positive and negative associations will cancel out each other.

#### 2.4.2 Permutation size and method

Permutations have been widely used in microarray data analysis to establish the null distribution of the test statistics [4,5,36]. The number of permutations chosen depends on statistical assumptions and the inferential goal and procedure. The aggregation method assuming the same null for all gene sets requires much fewer (1,000) permutations [4] than would a gene set specific null (10,000) [5]. However, the same null assumption does not always hold and when computationally affordable, we prefer to use gene set specific null [5]. In this study, we use gene set specific null approach with only 1000 permutations due to computational restrains. Using the example shown in the manual of *R *package *SAFE *[37] in Bioconductor [38] released with [5], we observe that the Yekutieli-Benjamini's resampling based FDR estimate [39] for a specific set [32] can range from 0.09 to 0.38 with different random seeds and a permutation size 1000. This illustrates the need to use a large number of permutations when setting the threshold based on a quantity requiring a good estimate of the p-value and motivated us to check whether permutation number 1000 is large enough in our study.

In our study, we bounded FDR rather than providing the set-specific FDR. With a relatively large threshold *p*_0 _(0.05 and 0.1), the compound-tissue-wise p-value estimation does not require high precision. We checked the reproducibility of our results with another 1000 permutations using a different random seed (Table 2). The identified gene sets from two runs are quite consistent except for *A*_*B*_(0.05) where the identified sets marginally satisfy the selection criterion.

**Table 2:** The number of identified sets and overlaps of two runs.

	Random seed 1	Random seed 2	Overlap
|*A*_*L*_(0.05)|	38	37	36
|*A*_*B*_(0.05)|	2	5	2
|*A*_*LB*_(0.05)|	0	0	0
|*A*_*L*_(0.1)|	74	70	68
|*A*_*B*_(0.1)|	28	30	27
|*A*_*LB*_(0.1)|	5	5	5

We randomly permuted the outcome variable ALT to generate the null distribution of ES. However, for a particular compound, samples taken at the same time point shared one control group. This raises the possibility that the observed association between ALT and gene expression might be influenced by the resulting statistical dependency. To evaluate this possibility, we redid the permutation while keeping the dependency structure. For example, in case of compound 1,4-dichlorobenzene, there are 3 dose levels and 3 time points, 4 replicates for each dose-time combination. The 12 samples for each time point sharing the same controls comprise a group. We randomly matched groups of ALT levels with groups of gene expression levels. There are a total of 3! = 6 possible group matches. In each matched group of 12 samples, we then randomly permuted ALT levels. Among the eight compounds, two did not have balanced subgroups for this type of permutation. We thus used only the other 6 compounds and ran this two-stage permutation 1000 times to generate 1000 "null" data sets per compound per tissue.

Since we only have 6 compounds in the new permutations, we cannot apply the "6 of 8" criterion to identify associated sets in the new permutation results. However, the Pearson correlation and Spearman rank correlation of *P *(and *Z*) values between the two permutations in each compound and tissue are all higher than 0.96, indicating the strong consistency of results between the two different permutation strategies. Thus our identified sets based on p-values would be almost the same with the two permutations methods.

## Conclusion

We present an inferential framework for selecting gene sets that are associated with a continuous phenotypic endpoint for microarray data with multiple categories of experimental conditions. We proceed in three steps: 1) compute gene specific *R*^2 ^between gene expression and endpoints for each individual category; 2) score the association of sets of genes with endpoints based on the gene specific association measurements; 3) combine category specific inference results to identify sets of genes that are associated with endpoint in most of the categories. When the phenotype is binary, many statistics used for identifying differentially expressed genes can be used in step 1. However, these statistics might not work well for continuous and non-linear/non-monotonic relationships between endpoint and gene expression. Based on natural cubic spline regression, our proposed *R*^2 ^not only captures non-linear associations between endpoint and gene expression but also accommodates any existing linear association between the two variables. In step 2, we adapt the framework of GSEA/SAFE using our *R*^2 ^as the association measurement. The combination of steps 1 and 2 can be regarded as a special case of the GSEA/SAFE procedure in a generalized sense. In step 3, we combine the results from multiple categories for each gene set and give a conservative FDR upper bound. This step depends on a subjective choice of threshold that can be tailored to the inferential goal. Different thresholds would result in different numbers of significant sets.

Assuming that the experimental information is fully represented by gene expression levels (Equation 1), we calculate the gene specific *R*^2 ^separately for each category. For some genes, this assumption might be violated if the curves relating gene expression level and endpoints are different for the same category at different experimental conditions. In this case, a pooled approach inside each category might yield reduced *R*^2 ^values. However, limited sample size of the category might not allow splitting the data into subcategories. Also, the curve (*X*_*ij*_, *Y*_*i*_) itself is driven by the experimental conditions, e.g., time and dose. If we fix all these factors, then pairs (*X*_*ij*_, *Y*_*i*_) may distribute around an average point and we would not be able to capture the main association trend.

We use the quantity *R*^2 ^to screen strong associations between endpoint and gene expression levels rather than to select the best model for predicting the endpoint from the gene expression level. Since the spline's degrees of freedom is fixed, it is not necessary to use a penalty term to control over-fitting. Finer model adjustment for purposes beyond screening can be considered after associated gene sets are identified.

We illustrate our method using the NCT compendium data in which the expression values from 20,500 genes in both rat liver and blood were analyzed at 3–4 dose levels and 3 time points for 8 hepatotoxicants. We are interested in identifying pre-defined gene sets that are associated with liver injury indicated by the ALT activity level in blood for most of the 8 hepatotoxicants. For this compendium data, we did observe non-linear association between ALT and gene expression. This might be due to the fact that ALT level increases as the degree of liver injury increases and, on the other side, the gene expression levels can be either up-regulated or down-regulated.

In the data example, we fixed 4 inner knots at 20%, 40%, 60%, and 80% quantiles of the gene expression. This model space was selected by manually examining scatter plots between gene expression levels and ALT levels. We believe that this model space is rich enough to account for biologically plausible non-linear/non-monotonic trend. If extra degrees of freedom are desired, a graph similar to Figure 2 can be used to evaluate the benefits. Although our proposed *R*^2 ^can capture non-linear associations between ALT and gene expression and lead to biologically meaningful inferences, computational feasibility restricts us from considering more complicated models, e.g., with two or more genes jointly in the model. While a high *R*^2 ^indicates strong association, a low *R*^2 ^does not always imply no association. Also, weak association may not be well captured by *R*^2^. However, this concern is alleviated in GSEA/SAFE procedure, because the method is designed to accumulate the weak local signals from individual genes into a stronger global signal at gene set level.

We compare the *R*^2 ^from a non-linear model and a linear model in Figure 2 to illustrate the need for using a non-linear model. Further performance comparison based on identified sets is difficult without knowing the true state of nature of whether a specific gene set is associated with the phenotypic endpoint. Also, we believe that in our case, it will not provide a very helpful reference to pursue a computational simulation, which has to include arguable assumptions that expressions of genes from one pathway follow some convenient multivariate distribution (e.g., multivariate normal distribution) and the outcome variable follows a skew distribution. As shown in the additional file 1, section 1, the null distributions of *R*^2 ^will be very sensitive to these assumptions.

In summary, we describe a framework of GSEA/SAFE for microarray data in which the gene expression data were generated under multiple categories of experimental conditions and the phenotypic endpoint was continuous. The proposed association measure *R*^2 ^successfully captured non-linear trends between the gene expression levels and endpoint. The *R*^2 ^was then incorporated into the GSEA/SAFE procedure in per category analysis. The usage of *R*^2 ^has the advantage over the t-statistics or Pearson correlation in identifying the gene sets with both genes positively and negatively correlated to endpoints. Inference across categories serves to identify gene sets, and the corresponding functional pathways whose alteration plays a role in related biological mechanism. Our method is general and can be applied to GSEA/SAFE analysis of microarray data with other continuous phenotype or multiple GSEA/SAFE analyses.

Finally, it is important to note that, in GSEA/SAFE analyses, global statistics is usually designed to test whether the distribution of local statistics within a gene set is different from that of genes outside the gene set. Proper local statistics and global statistics should be selected carefully to avoid the situation where a gene set is statistically significant but actually clusters in a region of weak association in the gene list and is off biological interest. In this article, we have used unsigned local statistics *R*^2^, signed global statistics and one-side p-value to eliminate this possibility.

## Competing interests

The authors declare that they have no competing interests.

## Authors' contributions

RL and LL conceived and designed the study and RL performed all analyses. SD prepared the rat gene set data. RI, AH, and GB provided biological interpretation of the results. RL and LL wrote the paper.

## Supplementary Material

Additional file 1 **Supplement: Gene Set Enrichment Analysis for non-monotone association and multiple experimental categories.** The file includes three sections: 1, the exploration on distribution of *R*^2 ^under permutation; 2, gene set data preparation; 3. identified gene sets and biological interpretation. Click here for file

Additional file 2 **Sets and Expression.** The intermediate analysis results of NCT compendium data set. Sets directory contains lists of identified gene sets under different across-compound (category) selection criteria. Compound specific p-values, set size, etc are provided. Expression directory contains extracted expression data for respective identified gene sets. Click here for file

Additional file 3 **Heat maps of sets in *A*_*LB*_(0:1).** Heat maps of gene sets in *A*_*LB*_(0:1). Both liver and blood data are presented for each identified set. Alt level is included after log10 transformation. Click here for file

Additional file 4 **Associations between genes in the identified set *glycolysis and gluconeogenesis *for liver data.** Figures that show associations between genes in the identified set *glycolysis and gluconeogenesis *for liver data. All 8 compounds are presented, including *monocrotaline *(Figure 3 in the article). Click here for file
