# Supplementary material for: Gene set enrichment analysis for non-monotone association and multiple experimental categories
Source: BMC Bioinformatics. 2008 Nov 14;9:481. doi: 10.1186/1471-2105-9-481 (PMC2636811; doi:10.1186/1471-2105-9-481)
Supplement: Additional file 1 — Supplement: Gene Set Enrichment Analysis for non-monotone association and multiple experimental categories. The file includes three sections: 1, the exploration on distribution of R2 under permutation; 2, gene set data preparation; 3. identified gene sets and biological interpretation. [file 1471-2105-9-481-S1.pdf]

Supplement: Gene Set Enrichment Analysis for non-monotone association and multiple experimental categories

1 Method

1.1 Exploration on distribution of R^2 under permutation

We explore the distribution of R^2 under permutation to find a potentially less computationally intensive approach to generate the null distribution of R^2.

When outcome variable vector $\mathbf{Y}$ with length n comes from a spherical distribution with $Pr(\mathbf{Y} = 0) = 0$, the asymptotic distribution of R^2 is a beta distribution with parameters $\frac{m}{2}$ and $\frac{n-m-1}{2}$, where m is the rank of the predictor matrix (Muirhead, 1982, Theorem 5.2.2). In reality, outcome variable Y can be very skewed due to the heterogeneous experimental conditions, and the spherical distribution assumption of (Muirhead, 1982, Theorem 5.2.2) would be severely violated.

For illustration, NCT compendium data are used. we compare permutation based null distribution of R^2 with $Beta(5/2, (n-5-1)/2)$, where 5 is the degrees of freedom for the splines. Figure 1 shows the deviation of the observed permutation based null distribution of R^2 from the Beta distribution. Each point in the figure represents one gene. When $\mathbf{Y}$ comes from a spherical distribution, R^2 follows Beta distribution and all clouds should stay around $(1,1)$. Outcome variable $\mathbf{Y}$ is fixed in each panel of Figure 1 and the variation between genes indicates that when $\mathbf{Y}$ does not follow spherical distribution, the distribution shapes of gene expression levels also play a role in deciding the distribution of gene specific R^2, considering R^2 itself is a scale free measurement. Visually we can classify the shapes of clouds in Figure 1(a) and 1(b) into four categories indicated by four colors in figures. Figure 2 shows that density curves of ALT levels standardized to mean 0 and variance 1. The panels can again be visually classified into four categories, which are consistent with the classification in Figure 1(a) and 1(b). This indicates that the distribution of ALT also has an impact on the distribution of permutation based R^2. Generally, the mean of permutation based R^2 is close to that of the respective Beta distribution with ratio between 0.9 to 1.1 while the range of variance ratio is relatively large from 0.3 to 3. We thus conclude that the null distribution of R^2 depends on both gene expression levels and ALT levels and for our analysis, the Beta approximation is not adequate.

GSEA and SAFE procedure permutes arrays to generate null distributions of R^2 while keeping the correlations between genes. For each permutation, a vector of gene specific R^2 is sampled from the joint distribution of R^2s. Had non-resampling approach to gene specific R^2 been feasible, we

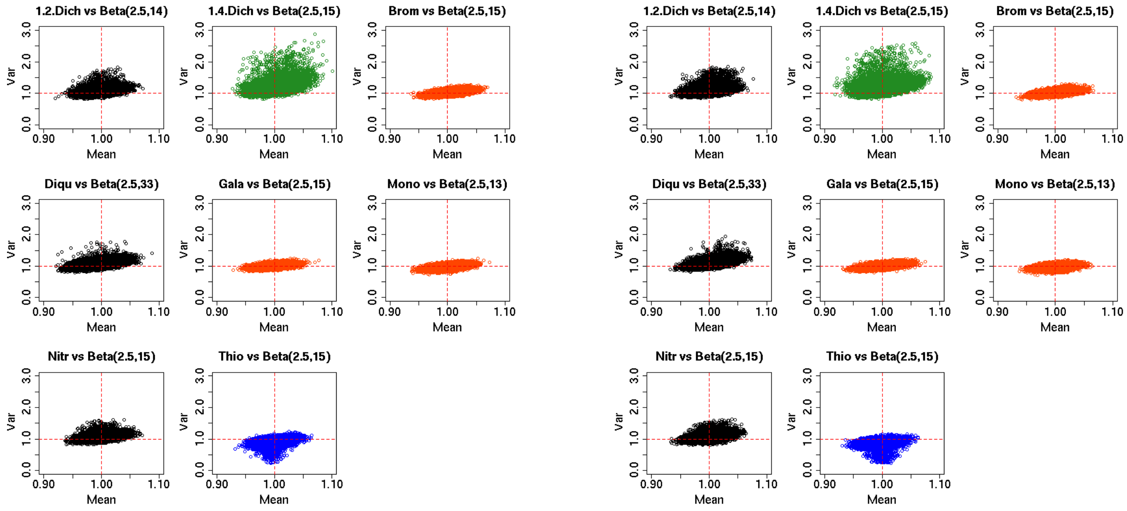

(a) Liver (b) Blood

Figure 1: Distribution of permutation-based R^2 vs Beta. Each point is for one gene. X-axis is the ratio of observed mean of permutation based R^2 to the mean of Beta. Y-axis is the ratio of the observed variance of permutation based R^2 to the variance of Beta.

need to further investigate how correlations between genes will impact the joint distributions of R^2, which will be used to calculate the global statistics.

2 Gene set preparation

We evaluated the association between ALT and the pre-established gene sets provided on GSEA's web site (http://www.broad.mit.edu/gsea/). Those gene sets were originally compiled from various sources including cytogenetic information, microarray studies, Gene Ontology, BioCarta and GenMAPP, etc., with presumable emphasis on cancer. Since the GSEA gene sets are for human genes, we first mapped the rat genes on the Agilent rat chip to the human genes in the GSEA sets. Here we focus on the functional category C2 which has 1137 functional sets. Among these 1137 sets, more than half are computationally predicted functional sets with hundreds of genes assumed based on previous microarray experiments. These computationally predicted sets are usually large and hard to interpret. Thus our subsequent analyses considered only sets from: BioCarta, GenMAPP, GO, SigmaAldrich, Signalling Alliance and Signalling Transduction KE, all of which are considered biological pathways/processes. A total of 466 gene sets (pathways) remain.

2.1 Data sets

(1) NCBI LocusLink database (LL_tmpl) ftp://ftp.ncbi.nih.gov/refseq/LocusLink/ARCHIVE/

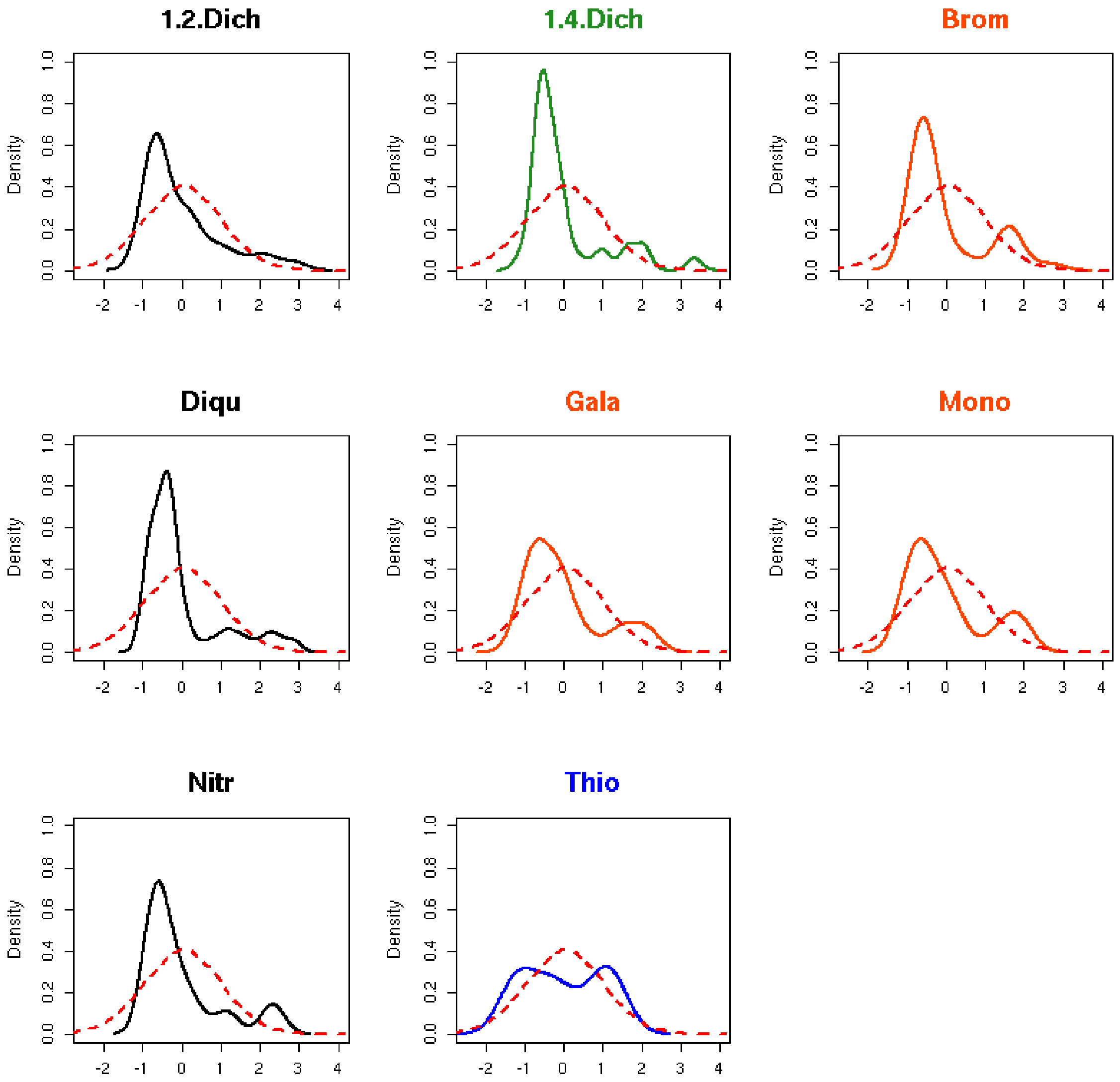

Figure 2: Density of standardized $\log_{10}$(ALT ratio) with mean 0 and variance 1. Red dashed line is the density function of Normal(0,1). All panels are in the same scale for comparison convenience.

This database contains 28,179 and 18,254 LocusIDs for human and rat, respectively. It serves as a reference database in the mapping process as it contains information about reference genes including refGene accession number(s), GeneBank accession number(s), protein accession number, official gene symbol and alias symbols.

(2) NCBI homologene database (build 46.1) ftp://ftp.ncbi.nih.gov/pub/HomoloGene

This database contains homologous genes in several species identified by protein accession numbers.

(3) UCSC hgTable (build 17) http://genome.ucsc.edu/cgi-bin/hgTables

This data set contains pair-wise reciprocal blastp results between human and rat protein sequences. We only considered pairs that have e-value as putative orthologous.

(4) Orthologous pairs from TIGR http://www.tigr.org/tigr-scripts/magic/r1.pl

TIGR has mapped probes among several commercial chips through reciprocal blastp search. The orthologous pairs we used are the Agilent rat/Affymetrix human and Agilent rat/Agilent human.

2.2 Construct human/rat orthologous pairs

We used the LocusLink database as a connection among other data sources using different identifiers so that they can be cross-referenced. On the other hand, the homologene database will be considered de facto gold standard and a starting point for identifying true ortholog pairs and resolving conflicts/inconsistency among other ortholog pair data sources.

We started with the NCBI homologene database and extracted all 14,337 human-rat orthologous pairs identified by protein accessions. Using the accessions, we extracted all corresponding refSeq and GenBank accessions and official/preferred and alias gene symbols.

Next we sequentially examined the putative orthologous pairs from the other two data sources (UCSC and TIGR). Orthologous pairs in these two sources but not identified in the NCBI homologene database were added to our human/rat homologous table. This added another 5,285 human/rat ortholog pairs (identified by accessions, not necessarily unique). The final number of human/rat ortholog pairs is 19,622 which will be used for the next stage (available upon request). Each pair contains all known GenBank and refGene accessions and official/preferred/alias symbols for the gene.

2.3 Map rat genes to human gene sets

The human gene sets were downloaded from the MIT Broad Institute's web site:
http://www.broad.mit.edu/cancer/software/gsea_beta.

Table 1: The identified 5 sets "significant" in both liver and blood ($A_{BL}(0.1)$).

	Set Name	Set Size
1	PYRUVATE METABOLISM	42
2	GLYCOLYSIS AND GLUCONEOGENESIS	42
3	PROPANOATE METABOLISM	36
4	EIF2PATHWAY	7
5	TRANSLATION FACTORS	36

Here were the steps used in the mapping process:

Step 1: Take one human official gene symbol from the GSEA gene set and find the entry or entries in the homologous pair table that contains the human official gene symbol. Typically one entry is matched.

Step 2: For each matched entry, check to see if the corresponding rat identifiers (accessions and official/preferred symbols) match the symbols/ accessions on the Agilent rat chip. Accession(s) are first checked and if there is a match, the gene on the Agilent rat chip was then mapped to the human gene in the set. If not, the symbol(s) are then manually checked to make sure no ambiguity.

3 Identified sets and biological interpretation

Table 1 lists all 5 sets in $A_{LB}(0.1)$, which is also a subset of $A_L(0.1)$ and $A_B(0.1)$. Gene sets 1,2 and 3 are comprised primarily of enzymes that catalyze many of the basic steps in glycolysis and gluconeogenesis. In general these gene sets show an overall down regulation of gluconeogenesis and up regulation of glycolysis in the liver, especially for compounds such as bromobenzene, monocrotaline, and thioacetamide that produce a high level of injury by 24 to 48 hours. Sets 4 and 5 are comprised of genes involved with the initiation of protein synthesis and show a strong up-regulation at the 24 and 48 hour time points for most of the compounds. Overall, this pattern suggests that by 24 hours after initial compound insult the liver is up-regulating energy producing pathways such as glycolysis, to support subsequent repair processes that begin between 24 and 48 hours after exposure.

The sets in $A_L(0.05)$ are biological process/pathways in cellular metabolism (beta-alanine metabolism, fatty acid metabolism, glutathione metabolism, glycine/serine/threonine metabolism, hexose metabolism, purine/pyrimidine metabolism, propanoate metabolism, pyruvate metabolism and tryptophan metabolism), cell cycle regulation, DNA replication and repair, protein synthesis, fatty acid synthesis, and stress response. The transcriptional activity in those pathways points to a general stress response, in particular oxidative stress as indicated by an activation of glutathione synthesis. While the cells try to conserve energy by down-regulation of energy demanding processes they are also preparing for repair processes by boosting amino acid metabolism and DNA replication. This overall picture is very similar to the more specific response indicated by the top

five sets discussed above.

All eight hepatotoxic compounds caused liver injury. However, the mechanisms of acute hepatotoxicity might be different, as different compounds may target different populations of cells in the liver. To see how the compounds might relate to each other, we carried out the following analysis. Let $Z^l_{c,s} = \Phi^{-1}(P^l_{c,s})$, where $l = L, B$ and $\Phi(\cdot)$ is the cumulative distribution function of a normal distribution with mean 0 and variance 1. For computations, we used $\frac{1000P^l_{c,s}+1}{1002}$ instead of $P^l_{c,s}$ such that transformation $\Phi^{-1}(\cdot)$ can be applied to the sets with $P^l_{c,s}$ equal to 0 or 1. We computed the pair-wise Pearson correlation coefficient of the z-scores of the 466 gene sets between compounds for both liver and blood. In general, the compounds in liver (Figure 3) show more similarity and pattern than in blood. In Figure 3, as expected, 1,2- and 1,4-dichlorobenzene are relatively highly correlated as these two compounds are positional isomers. Since 1,4-dichlorobenzene is the least toxic compound, it is not surprising that its correlations with the other six compounds are low. Diquat shows the least correlation with any other compound. The pathology results indicate that diquat primarily affects endothelial cells Atkinson et al. (2001) with hepatocyte damage secondary to the anoxia. Although monocrotaline also targets endothelial cells, they act by different mechanisms with diquat causing lipid peroxidation of cell membranes Atkinson et al. (2001). The precise mechanism of monocrotaline toxicity is unknown but may in part be due to oxidation Baybutt and Molteni (1999). No strong correlation between monocrotaline and diquat was found in a similar microarray study Waring et al. (2001).

References

Atkinson, J. B., K. E. Hill, and R. F. Burk (2001, Feb). Centrilobular endothelial cell injury by diquat in the selenium-deficient rat liver. *Lab Invest 81*(2), 193–200.

Baybutt, R. C. and A. Molteni (1999, Sep). Dietary beta-carotene protects lung and liver parenchyma of rats treated with monocrotaline. *Toxicology 137*(2), 69–80.

Muirhead, R. J. (1982). *Aspects of Multivariate Statistical Theory*. John Wiley & Sons.

Waring, J. F., R. A. Jolly, R. Ciurlionis, P. Y. Lum, J. T. Praestgaard, D. C. Morfitt, B. Buratto, C. Roberts, E. Schadt, and R. G. Ulrich (2001, Aug). Clustering of hepatotoxins based on mechanism of toxicity using gene expression profiles. *Toxicol Appl Pharmacol 175*(1), 28–42.

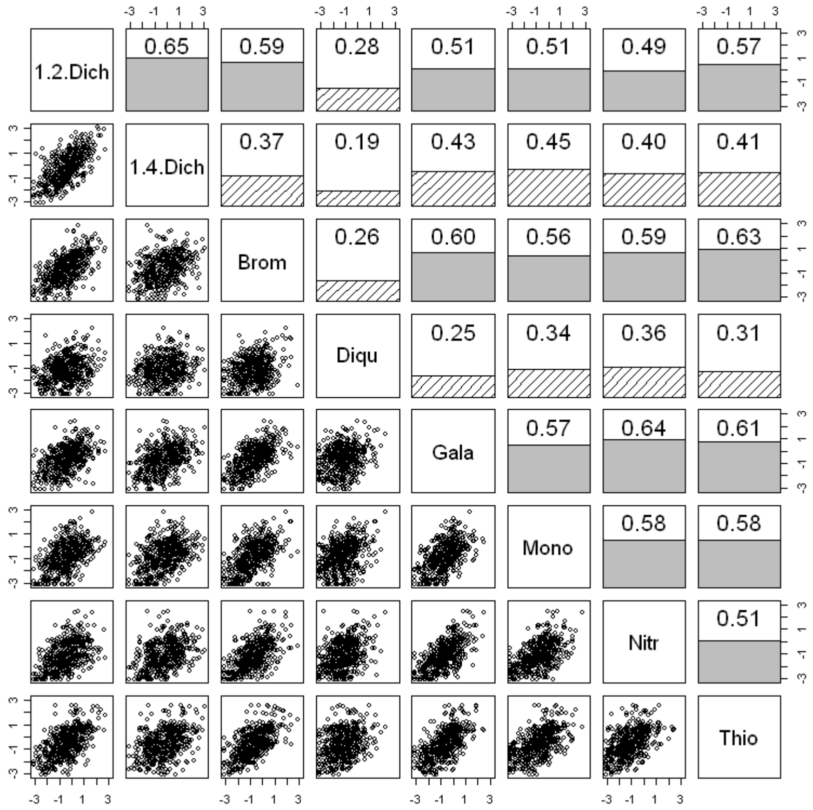

Figure 3: Pairwise scatter plot of Z-values of 466 sets in 8 compounds with simplified compound names listed in diagonal panels. In upper panels, the correlation coefficients between Z-values are shown. In each panel, a rectangle is drawn in height proportional to the absolute value of the correlation coefficient. Rectangles of relatively lower correlation coefficients comparing to the others are shadowed in slanting lines.
