# Supplementary material for: Gene set enrichment analysis for non-monotone association and multiple experimental categories
Source: BMC Bioinformatics. 2008 Nov 14;9:481. doi: 10.1186/1471-2105-9-481 (PMC2636811; doi:10.1186/1471-2105-9-481)
Supplement: Additional file 3 — Heat maps of sets in ALB(0:1). Heat maps of gene sets in ALB(0:1). Both liver and blood data are presented for each identified set. Alt level is included after log10 transformation. [file 1471-2105-9-481-S3.ppt]

## Slide 1
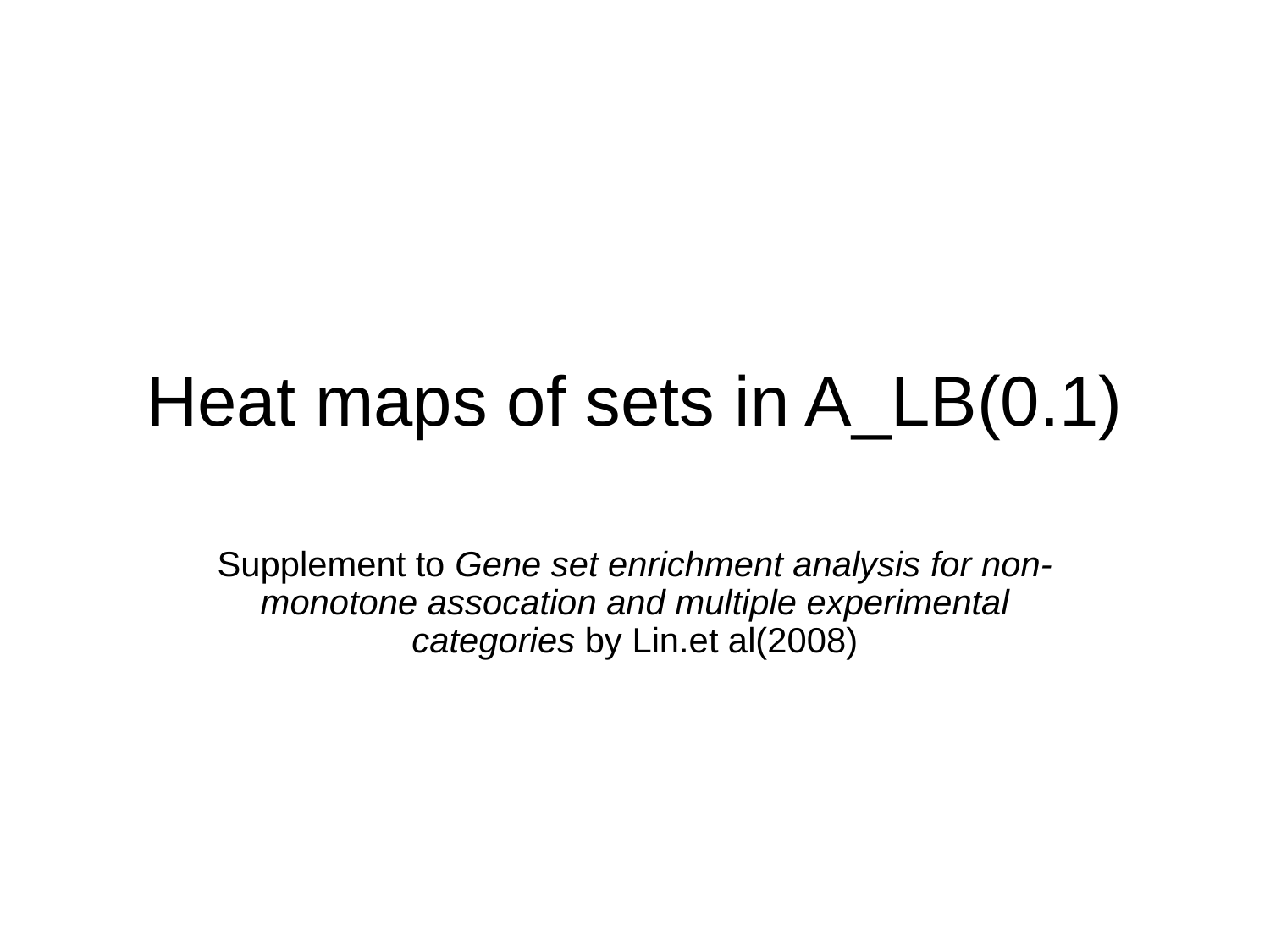

# Heat maps of sets in A_LB(0.1)
Supplement to Gene set enrichment analysis for non-monotone assocation and multiple experimental categories by Lin.et al(2008)

## Slide 2
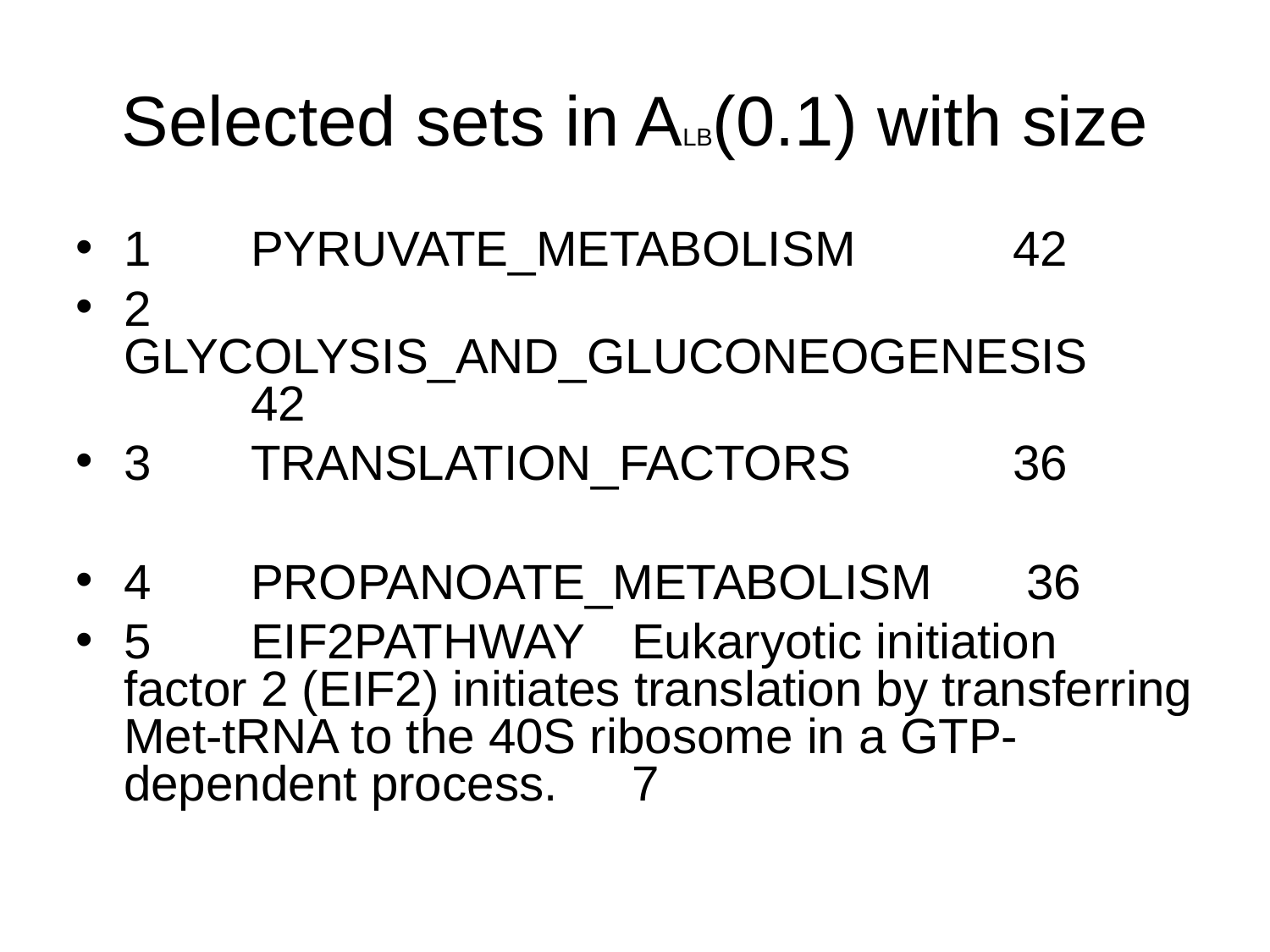

# Selected sets in ALB(0.1) with size
1	PYRUVATE_METABOLISM	 	42
2	GLYCOLYSIS_AND_GLUCONEOGENESIS	 	42
3	TRANSLATION_FACTORS	 	36
4	PROPANOATE_METABOLISM	 36
5	EIF2PATHWAY	Eukaryotic initiation factor 2 (EIF2) initiates translation by transferring Met-tRNA to the 40S ribosome in a GTP-dependent process. 	7

## Slide 3
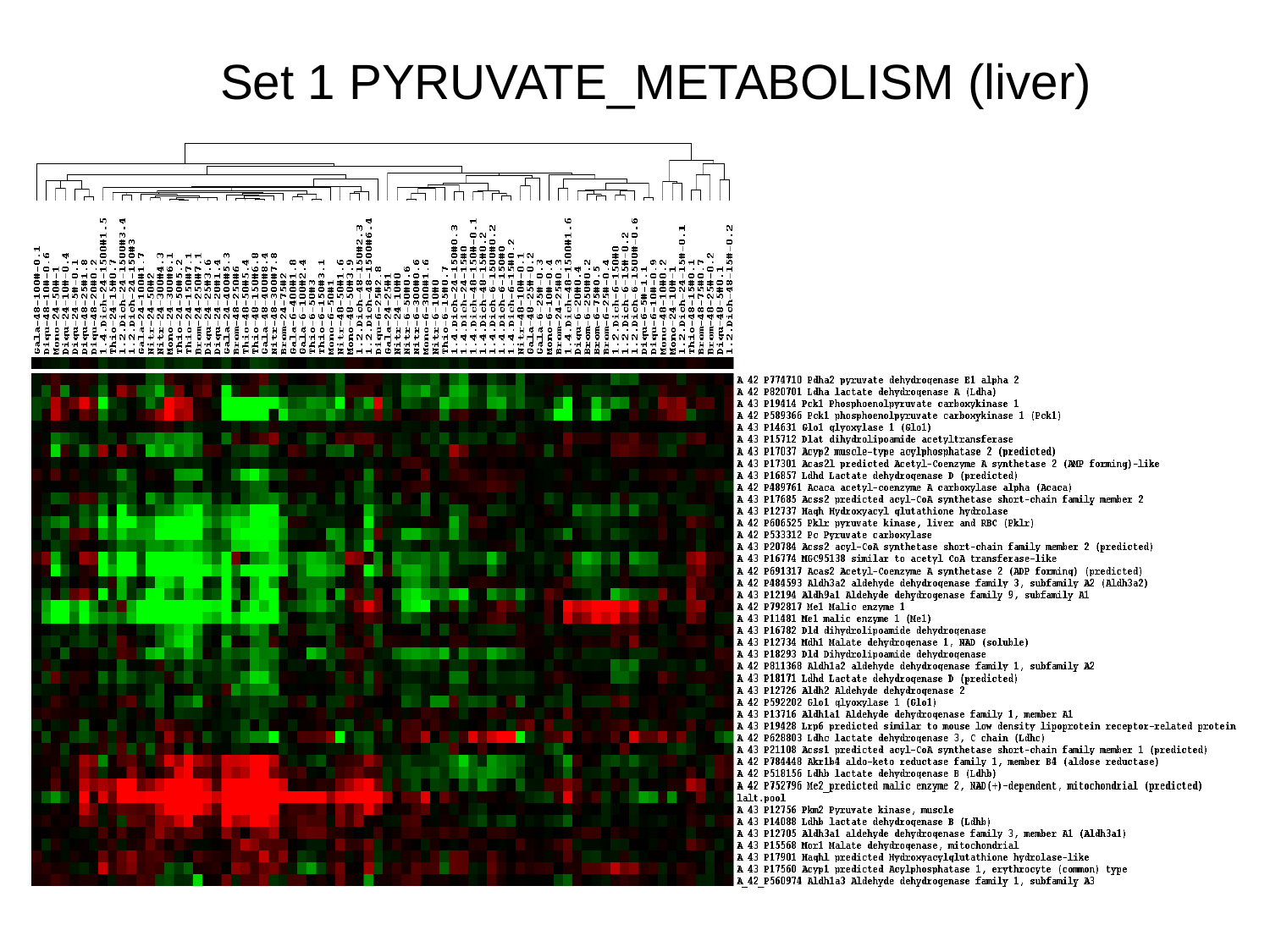

# Set 1 PYRUVATE_METABOLISM (liver)

## Slide 4
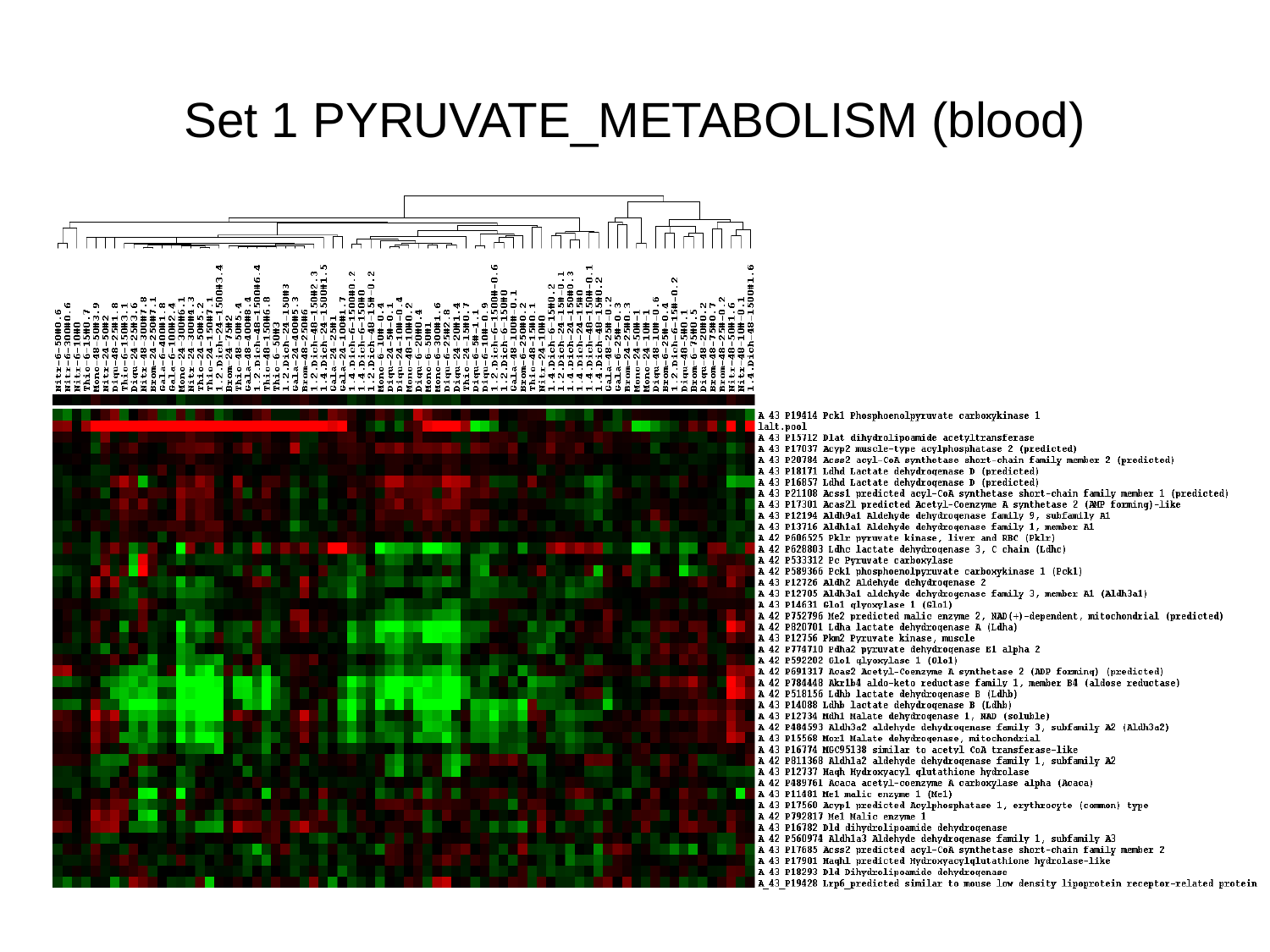

# Set 1 PYRUVATE_METABOLISM (blood)

## Slide 5
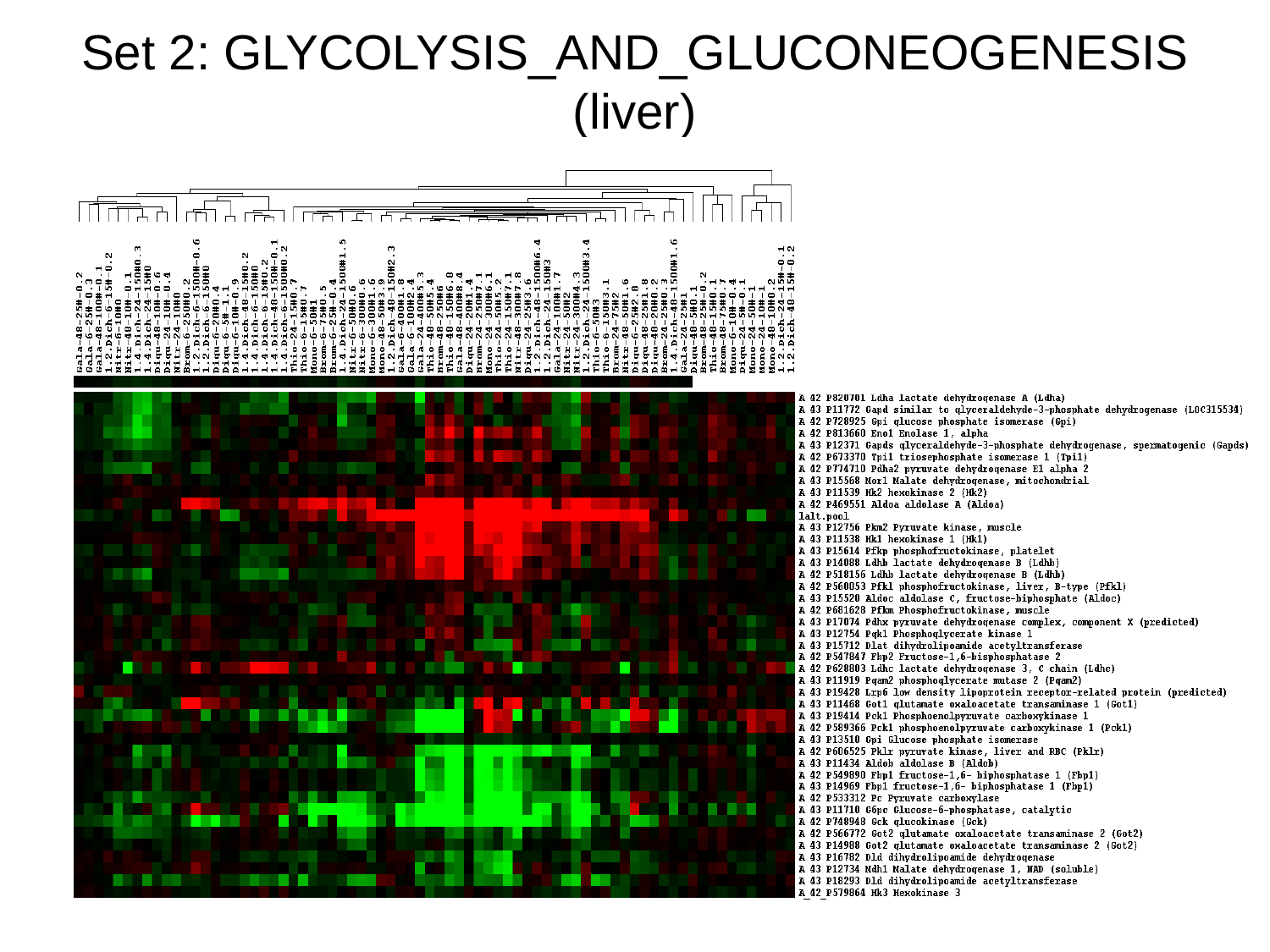

# Set 2: GLYCOLYSIS_AND_GLUCONEOGENESIS (liver)

## Slide 6
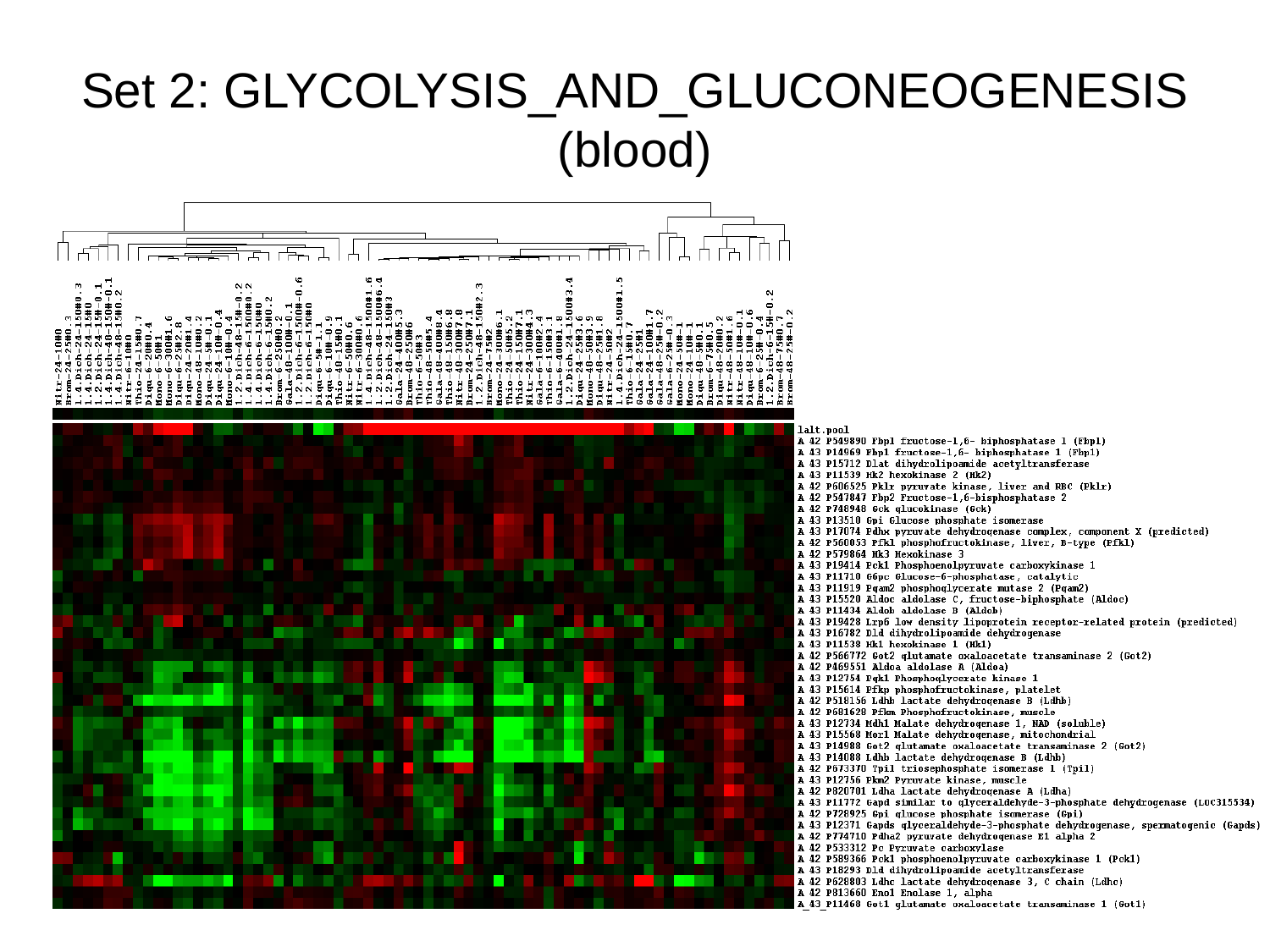

# Set 2: GLYCOLYSIS_AND_GLUCONEOGENESIS (blood)

## Slide 7
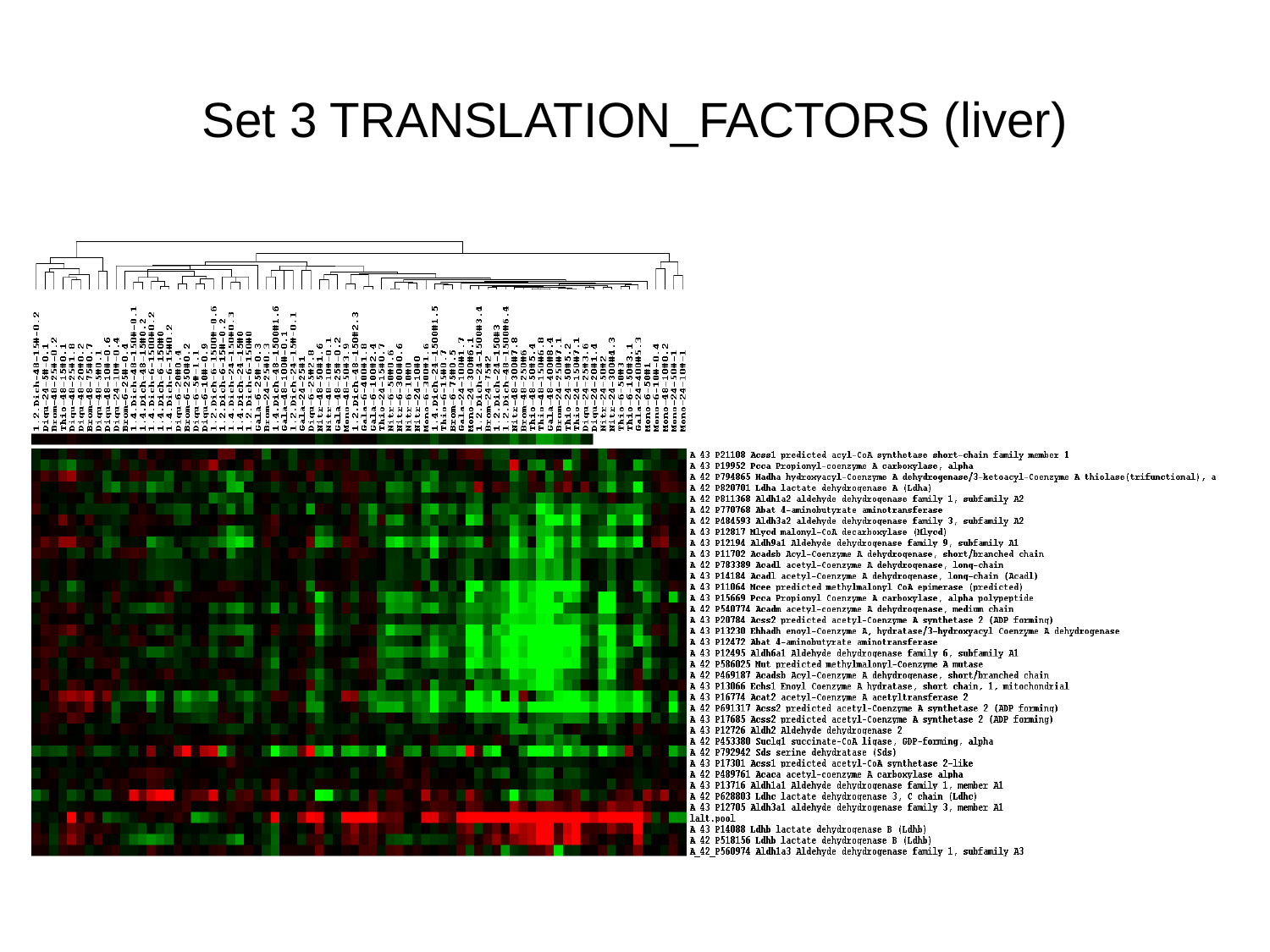

# Set 3 TRANSLATION_FACTORS (liver)

## Slide 8
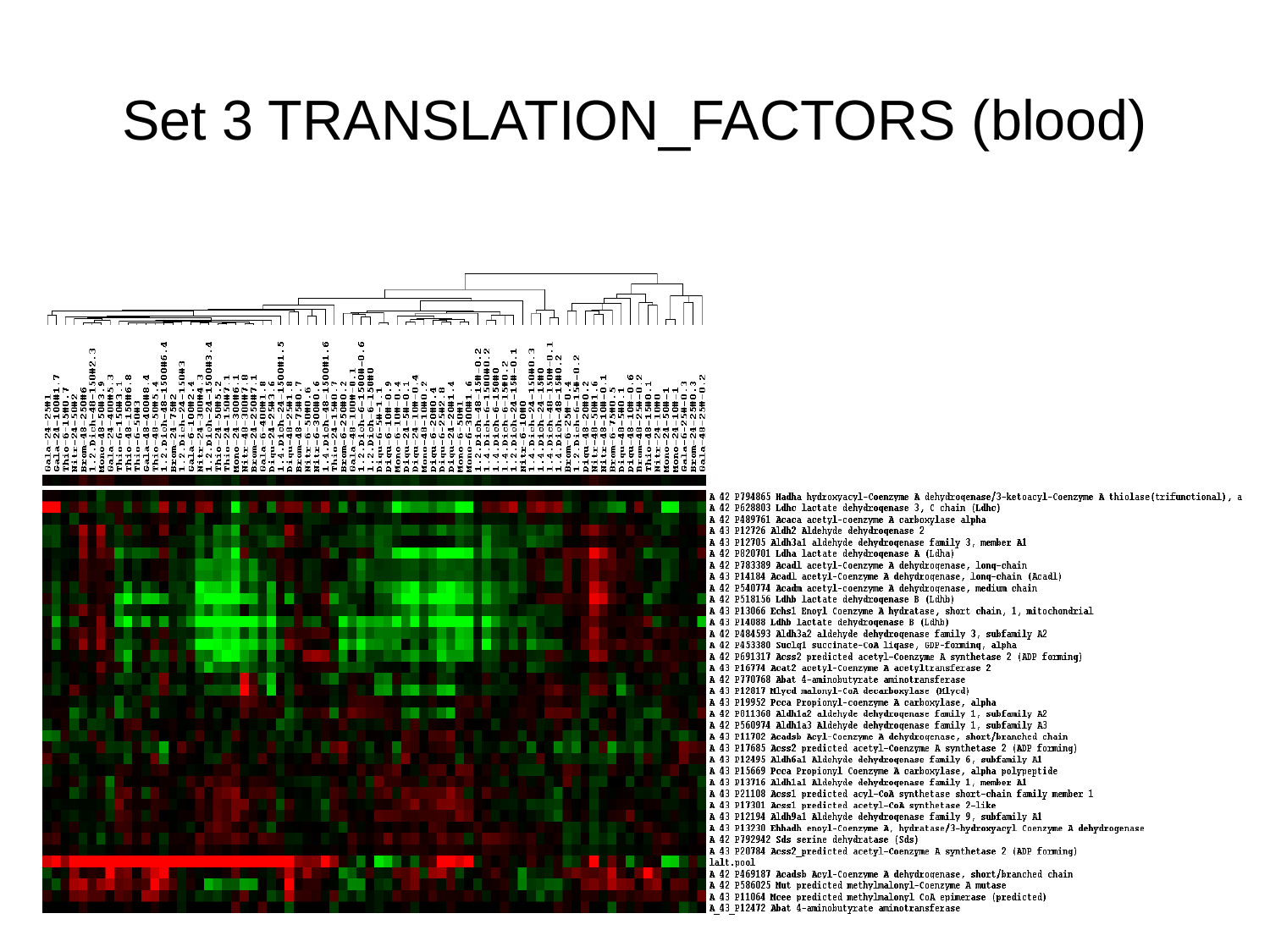

# Set 3 TRANSLATION_FACTORS (blood)

## Slide 9
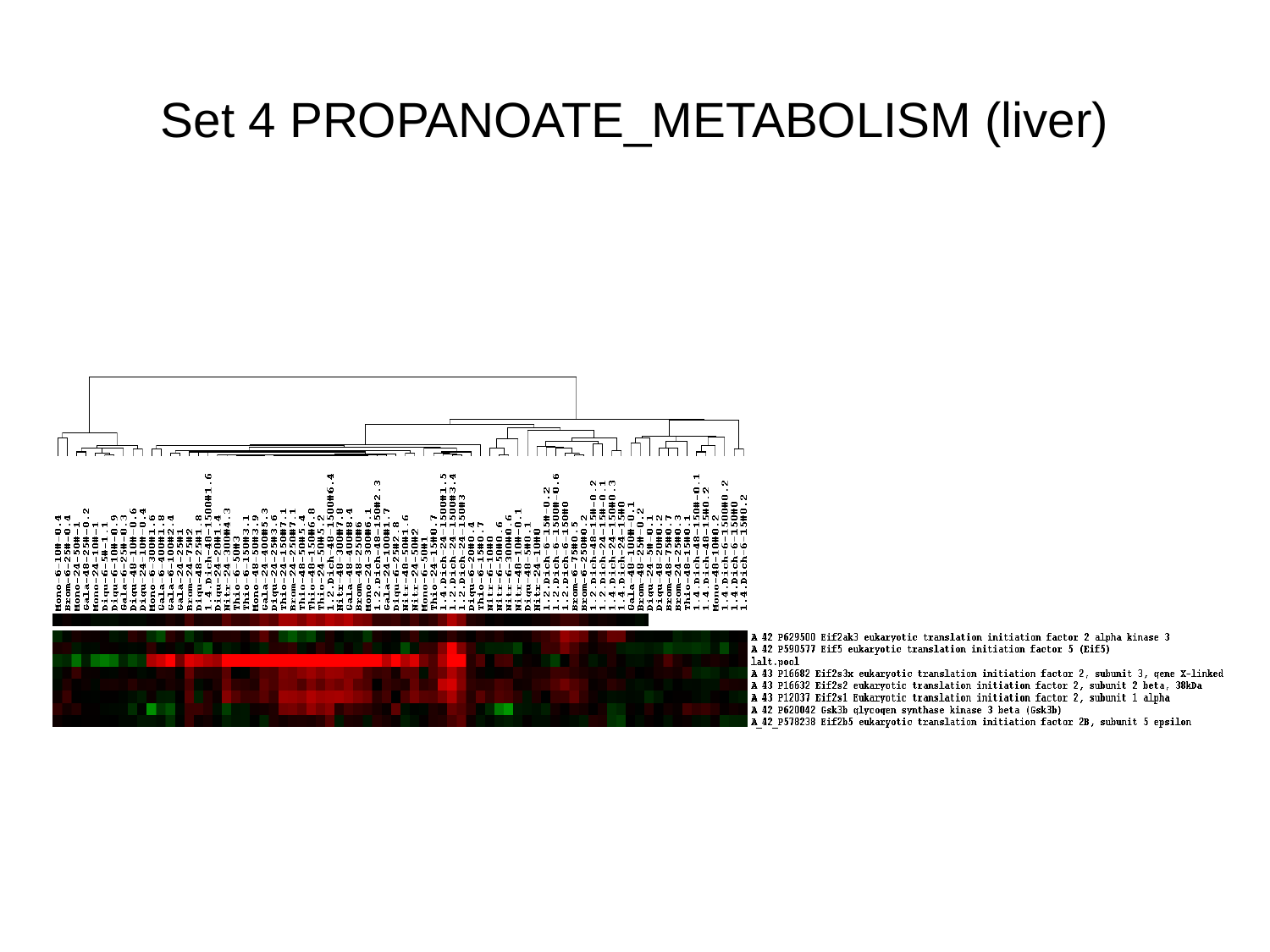

# Set 4 PROPANOATE_METABOLISM (liver)

## Slide 10
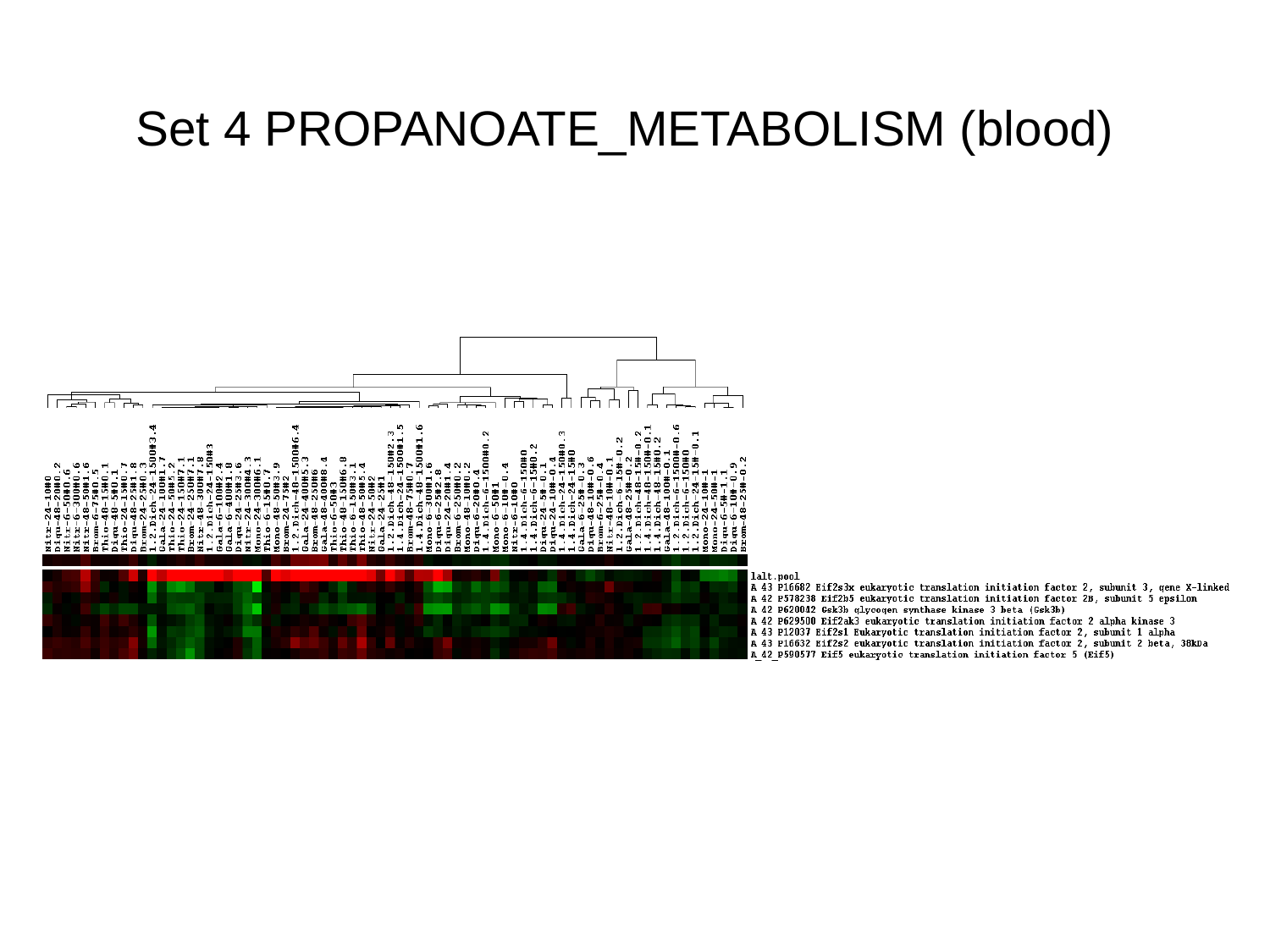

# Set 4 PROPANOATE_METABOLISM (blood)

## Slide 11
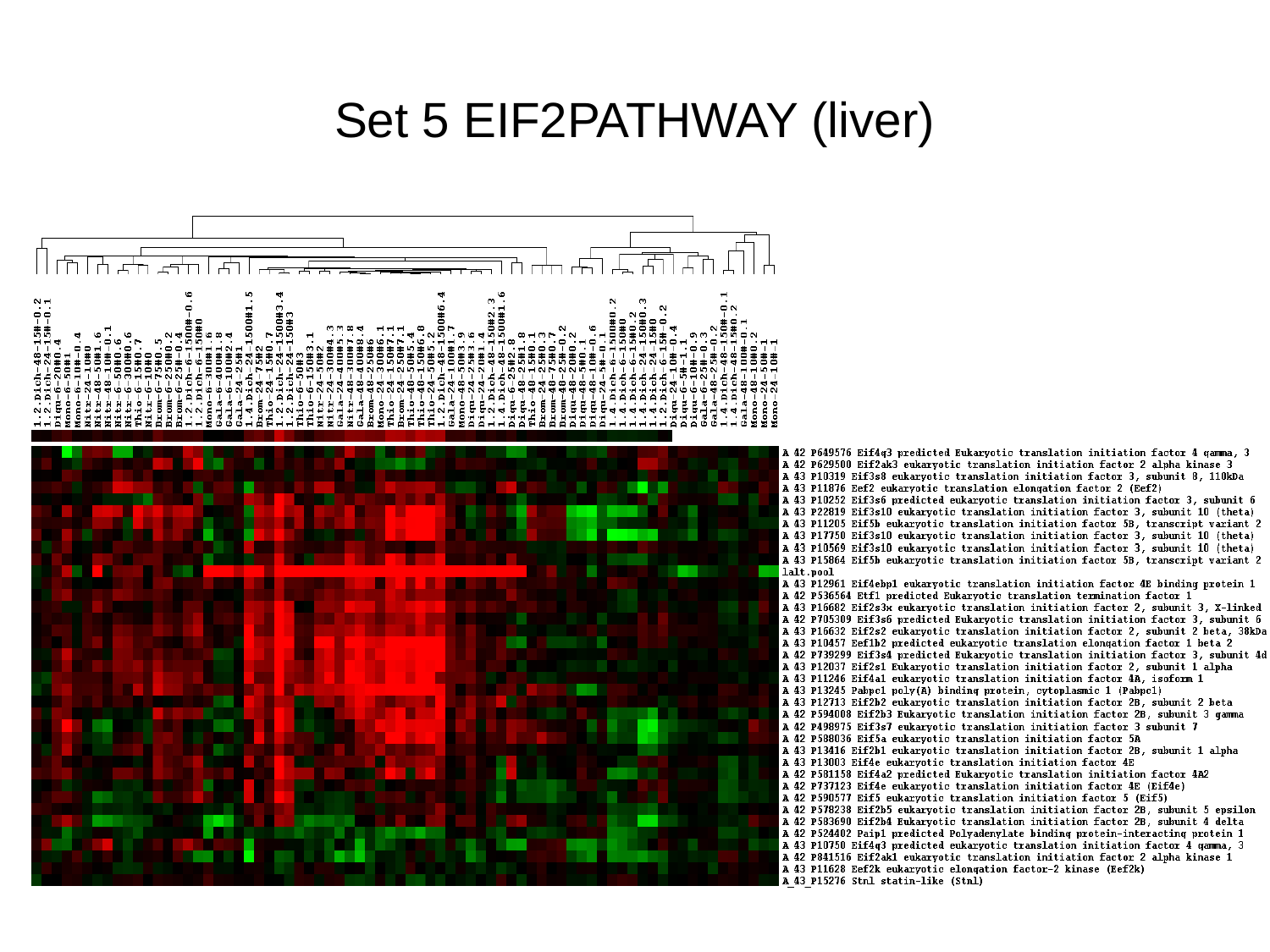

# Set 5 EIF2PATHWAY (liver)

## Slide 12
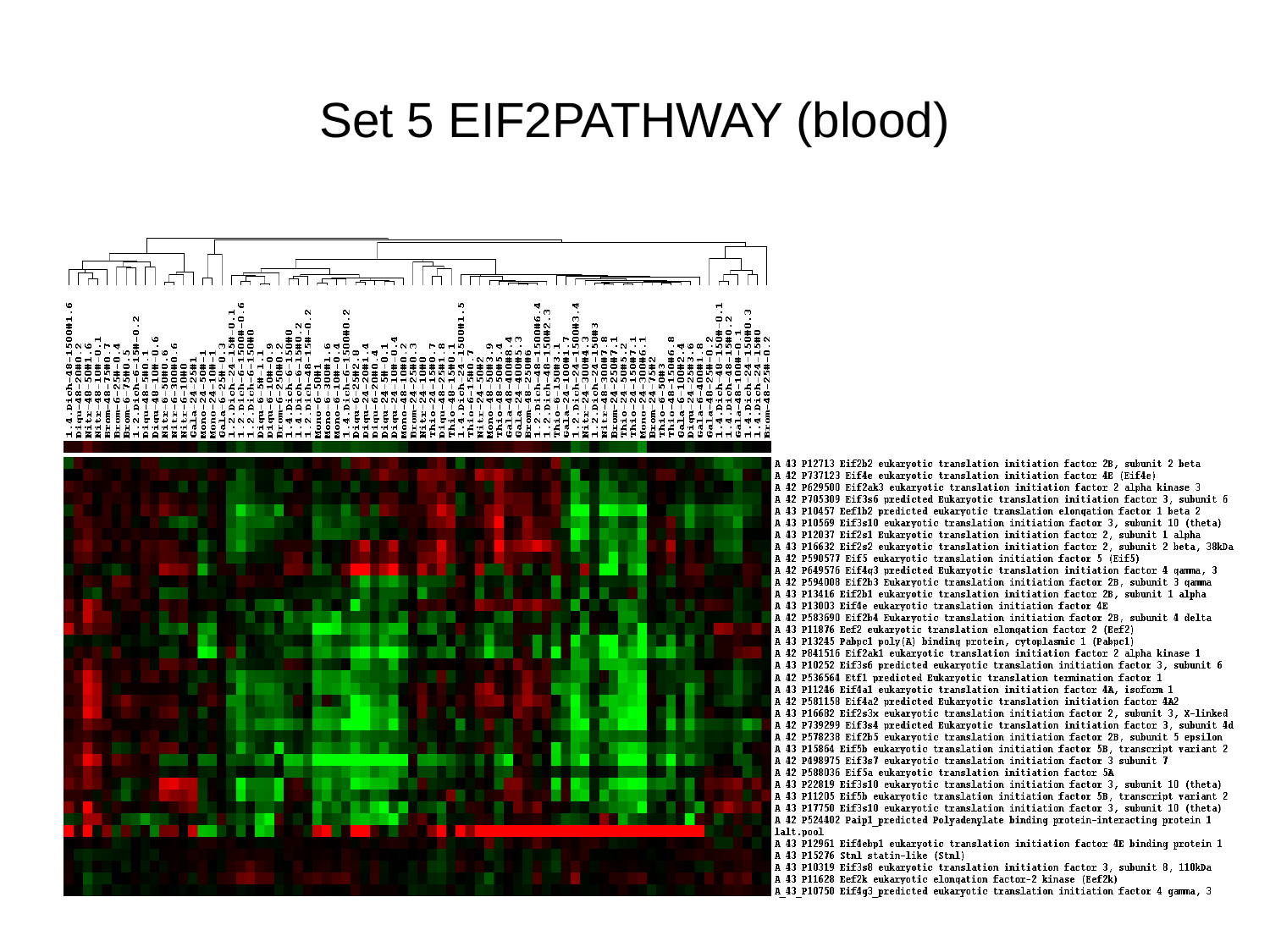

# Set 5 EIF2PATHWAY (blood)
