# Supplementary material for: Gene set enrichment analysis for non-monotone association and multiple experimental categories
Source: BMC Bioinformatics. 2008 Nov 14;9:481. doi: 10.1186/1471-2105-9-481 (PMC2636811; doi:10.1186/1471-2105-9-481)
Supplement: Additional file 4 — Associations between genes in the identified set glycolysis and gluconeogenesis for liver data. Figures that show associations between genes in the identified set glycolysis and gluconeogenesis for liver data. All 8 compounds are presented, including monocrotaline (Figure 3 in the article). [file 1471-2105-9-481-S4.pdf]

Associations between genes in the identified set

glycolysis and gluconeogenesis

for liver data

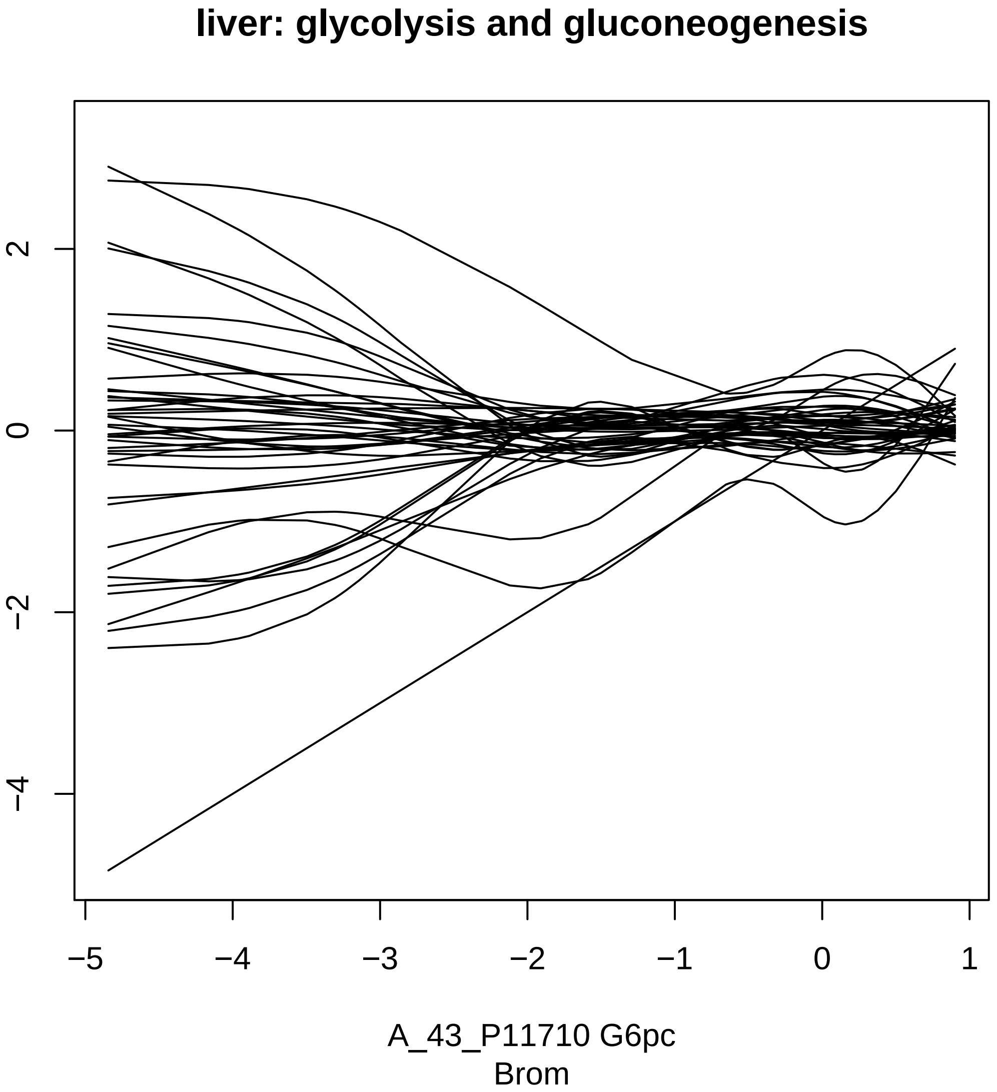

liver: glycolysis and gluconeogenesis
2
0
−2
−4
−5
−4
−3
−2
−1
0
1
A_43_P11710 G6pc
Brom

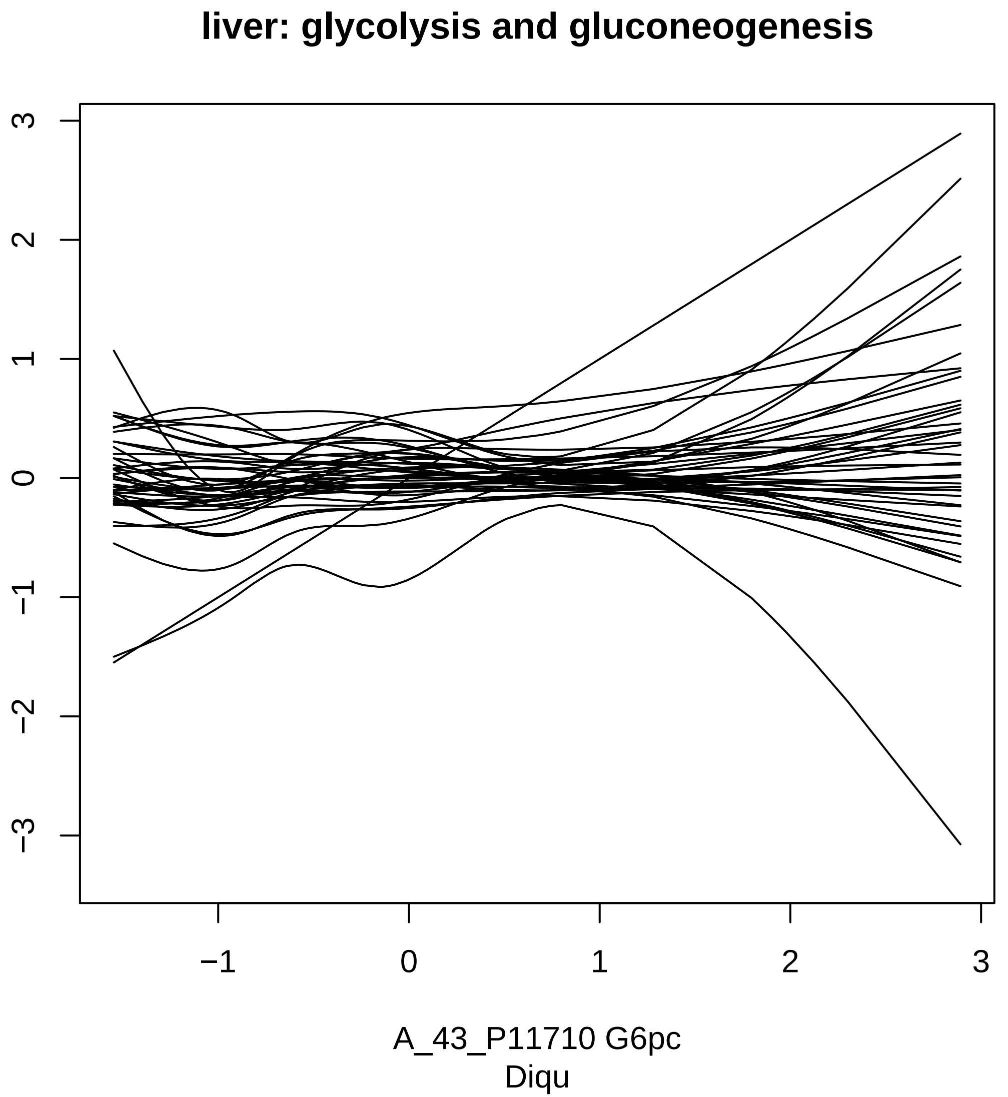
liver: glycolysis and gluconeogenesis
3
2
1
0
−1
−2
−3
−1
0
1
2
3
A_43_P11710 G6pc
Diqu

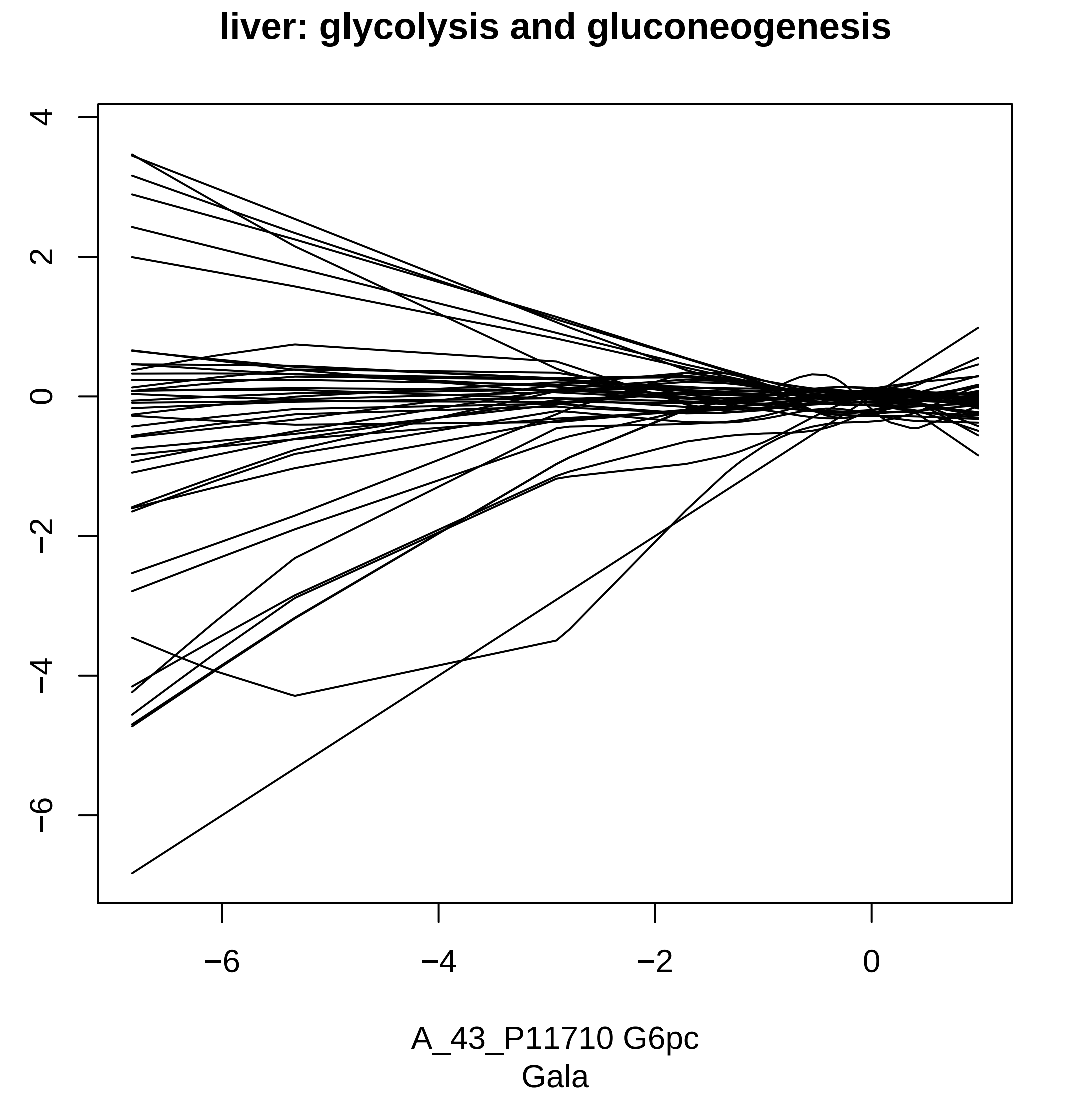
liver: glycolysis and gluconeogenesis
4
2
0
−2
−4
−6
−6
−4
−2
0
A_43_P11710 G6pc
Gala

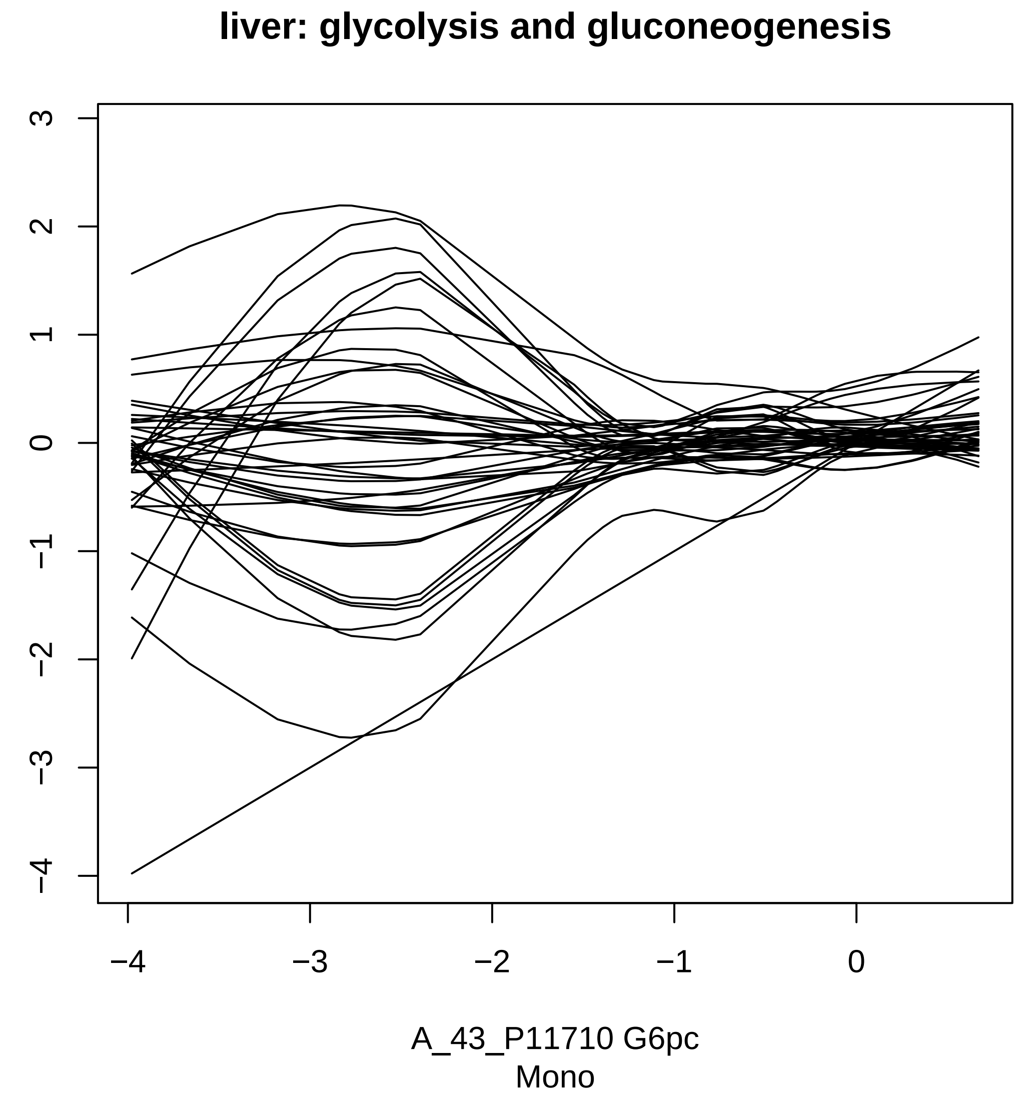

liver: glycolysis and gluconeogenesis
3
2
1
0
−1
−2
−3
−4
−4
−3
−2
−1
0
A_43_P11710 G6pc
Mono

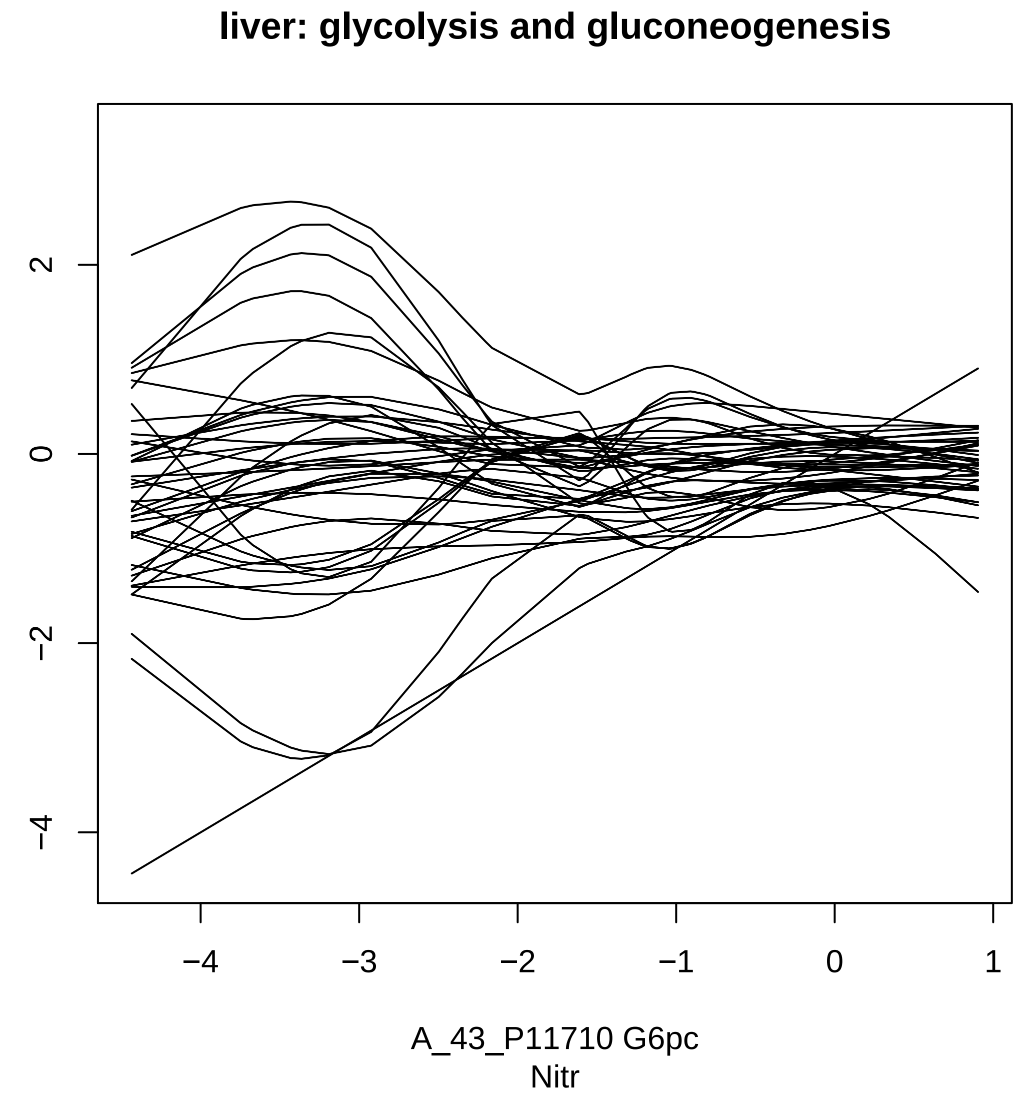
liver: glycolysis and gluconeogenesis
2
0
−2
−4
−4
−3
−2
−1
0
1
A_43_P11710 G6pc
Nitr

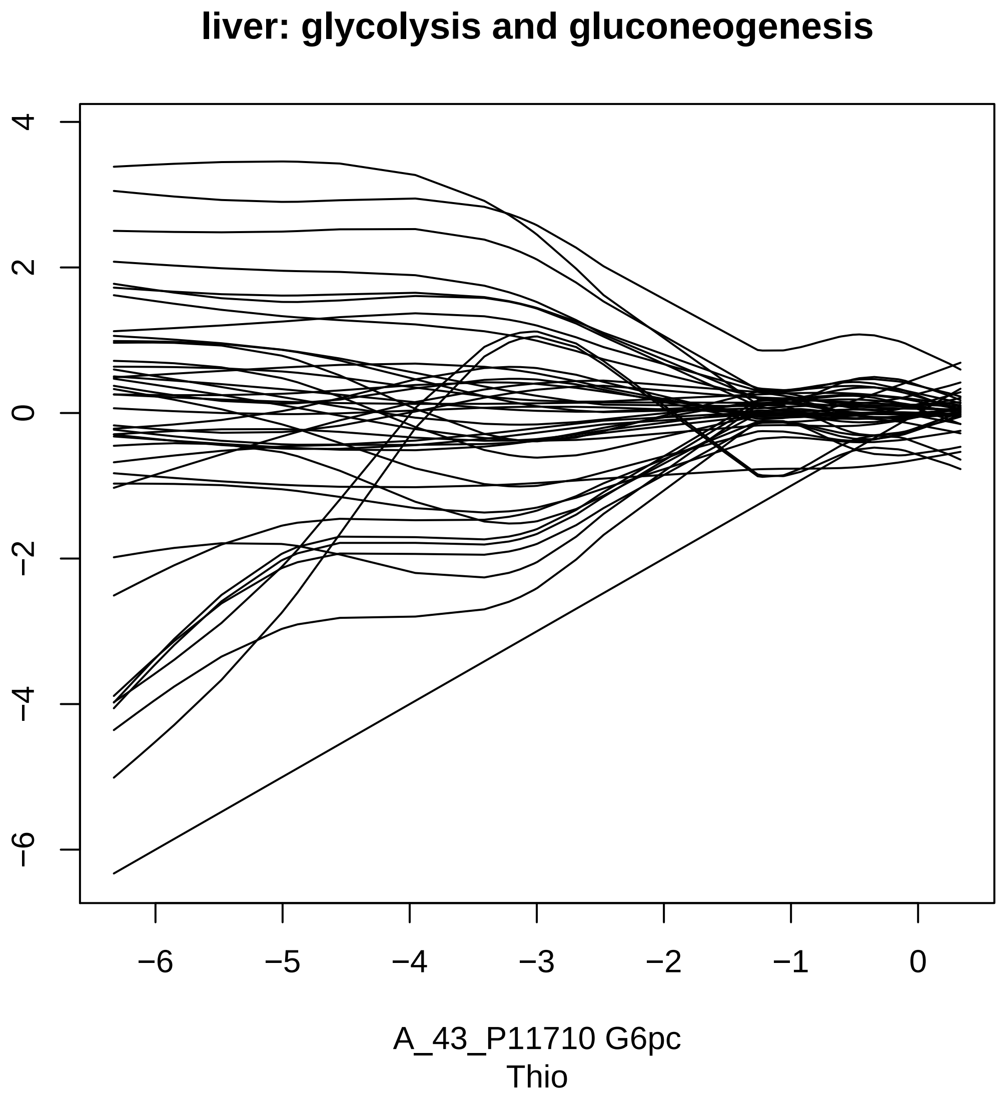

liver: glycolysis and gluconeogenesis
4
2
0
−2
−4
−6
−6
−5
−4
−3
−2
−1
0
A_43_P11710 G6pc
Thio

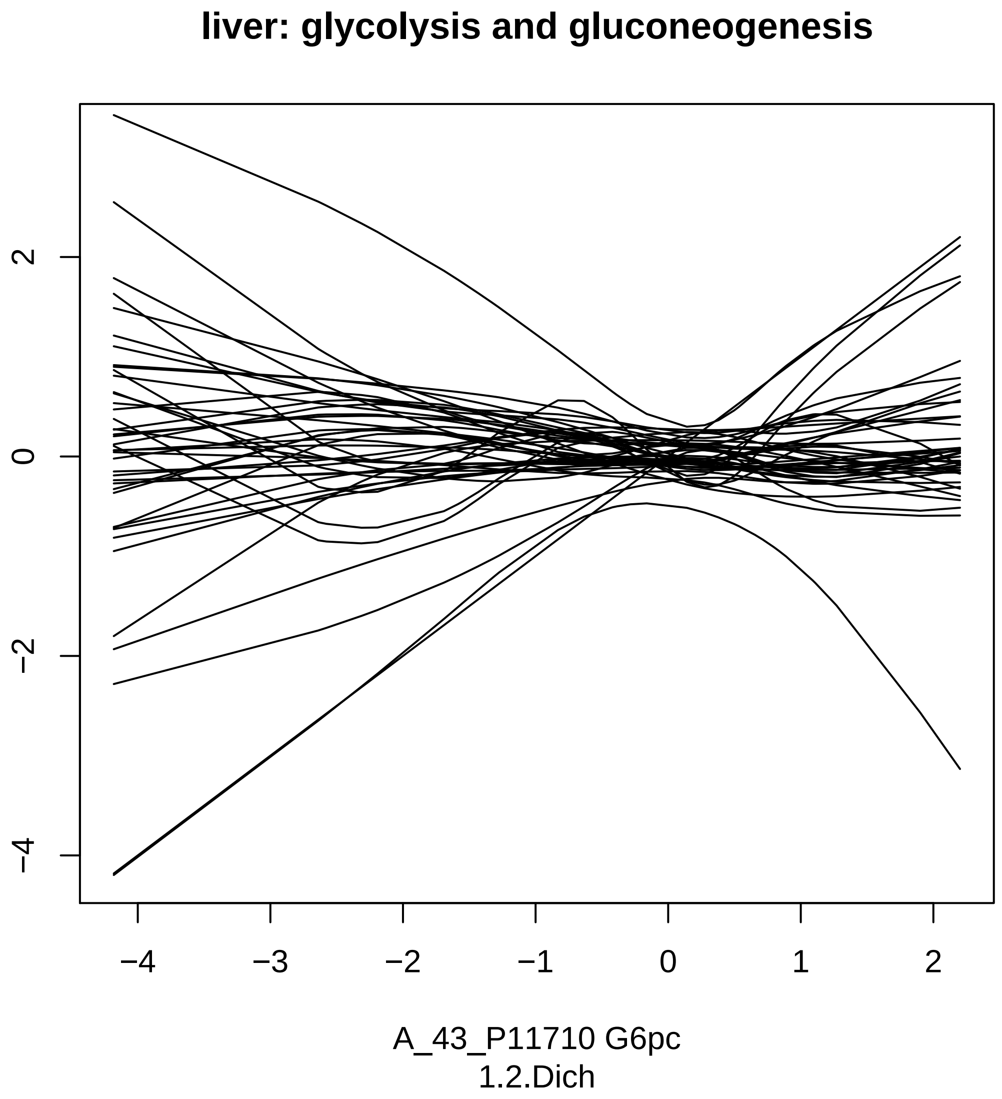
liver: glycolysis and gluconeogenesis
2
0
−2
−4
−4
−3
−2
−1
0
1
2
A_43_P11710 G6pc
1.2.Dich

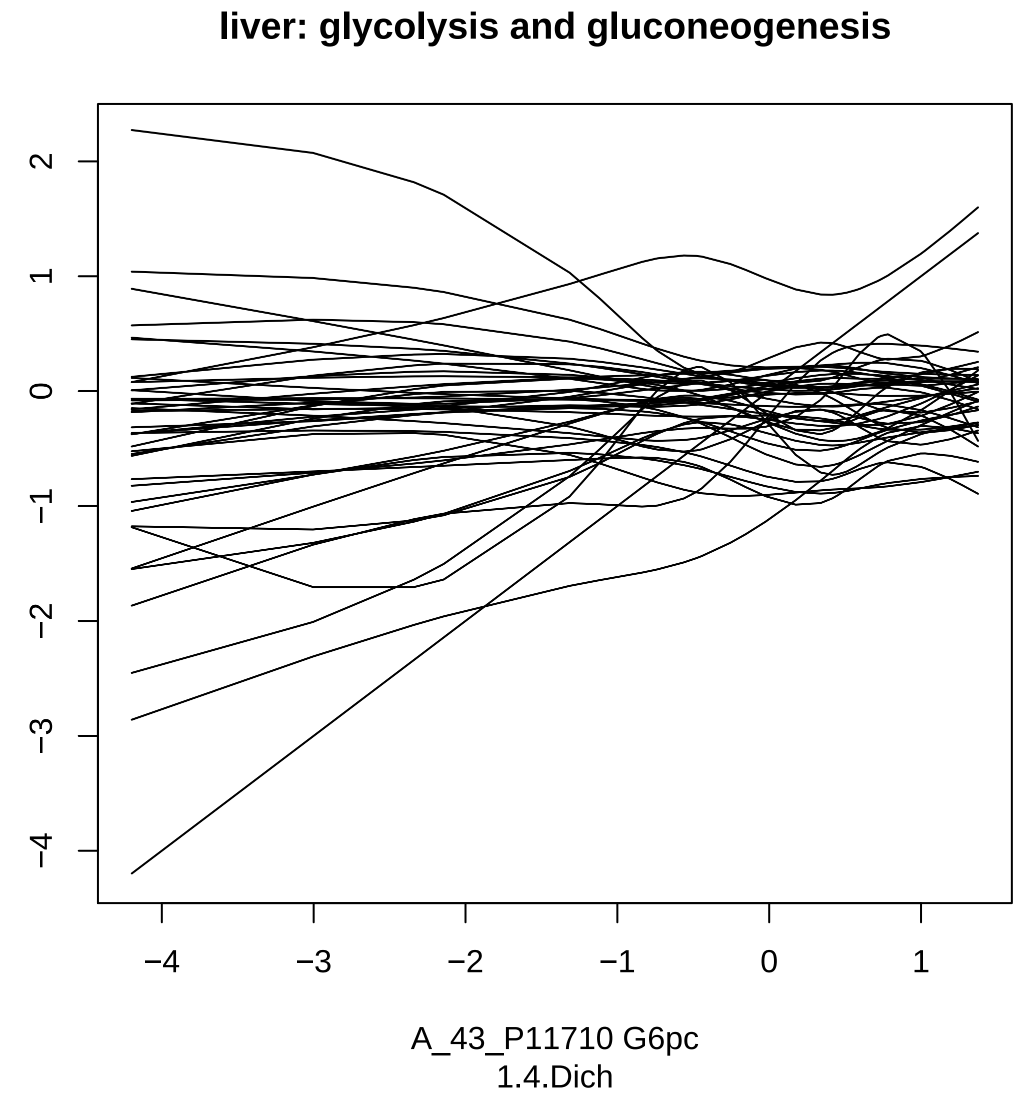

liver: glycolysis and gluconeogenesis
2
1
0
−1
−2
−3
−4
−4
−3
−2
−1
0
1
A_43_P11710 G6pc
1.4.Dich
